# Dietary Polyphenols Effects on Focal Adhesion Plaques and Metalloproteinases in Cancer Invasiveness

**DOI:** 10.3390/biomedicines12030482

**Published:** 2024-02-21

**Authors:** Raffaele Carrano, Martina Grande, Eleonora Leti Maggio, Carlotta Zucca, Riccardo Bei, Camilla Palumbo, Chiara Focaccetti, Daniela Nardozi, Valeria Lucarini, Valentina Angiolini, Patrizia Mancini, Francesca Barberini, Giovanni Barillari, Loredana Cifaldi, Laura Masuelli, Monica Benvenuto, Roberto Bei

**Affiliations:** 1 Department of Clinical Sciences and Translational Medicine, University of Rome “Tor Vergata”, Via Montpellier 1, 00133 Rome, Italy; raffaele.carrano@alumni.uniroma2.eu (R.C.); martina.grande@alumni.uniroma2.eu (M.G.); eleonora.letimaggio@alumni.uniroma2.eu (E.L.M.); carlotta.zucca@students.uniroma2.eu (C.Z.); camilla.palumbo@uniroma2.it (C.P.); chiara.focaccetti@uniroma2.it (C.F.); francesca.barberini.04@students.uniroma2.eu (F.B.); barillar@uniroma2.it (G.B.); cifaldi@med.uniroma2.it (L.C.); monica.benvenuto@unicamillus.org (M.B.); 2 Medical School, University of Rome “Tor Vergata”, Via Montpellier 1, 00133 Rome, Italy; riccardo.bei@students.uniroma2.eu; 3 Department of Experimental Medicine, University of Rome “Sapienza”, Viale Regina Elena 324, 00161 Rome, Italy; daniela.nardozi@uniroma1.it (D.N.); valeria.lucarini@uniroma1.it (V.L.); valentina.angiolini@uniroma1.it (V.A.); patrizia.mancini@uniroma1.it (P.M.); laura.masuelli@uniroma1.it (L.M.); 4 Departmental Faculty of Medicine and Surgery, Saint Camillus International University of Health and Medical Sciences, via di Sant’Alessandro 8, 00131 Rome, Italy

**Keywords:** focal adhesion plaques, polyphenols, integrins, FAK, cytoskeleton, matrix metalloproteinases, extracellular matrix, epithelial–mesenchymal transition, metastasis, cancer

## Abstract

Focal adhesion plaques (FAPs) play an important role in the communication between cells and the extracellular matrix (ECM) and in cells’ migration. FAPs are macromolecular complexes made by different proteins which also interact with matrix metalloproteinases (MMPs). Because of these fundamental properties, FAPs and MMPs are also involved in cancer cells’ invasion and in the metastatic cascade. The most important proteins involved in FAP formation and activity are (i) integrins, (ii) a complex of intracellular proteins and (iii) cytoskeleton proteins. The latter, together with MMPs, are involved in the formation of filopodia and invadopodia needed for cell movement and ECM degradation. Due to their key role in cancer cell migration and invasion, MMPs and components of FAPs are often upregulated in cancer and are thus potential targets for cancer therapy. Polyphenols, a large group of organic compounds found in plant-based food and beverages, are reported to have many beneficial healthy effects, including anticancer and anti-inflammatory effects. In this review, we discuss the growing evidence which demonstrates that polyphenols can interact with the different components of FAPs and MMPs, inhibit various pathways like PI3K/Akt, lower focal adhesion kinase (FAK) phosphorylation and decrease cancer cells’ invasiveness, leading to an overall antitumoral effect. Finally, here we highlight that polyphenols could hold potential as adjunctive therapies to conventional cancer treatments due to their ability to target key mechanisms involved in cancer progression.

## 1. Introduction

The focal adhesion plaques (FAPs) are integrin-containing protein complexes consisting of different classes of molecules that connect the cytoskeleton with the extracellular matrix (ECM). This whole system is extremely dynamic, and the continuous turnover and changes in conformation of its components, due to transient modifications such as protein phosphorylation and dephosphorylation, affect the behavior of the cells themselves [1]. Focal adhesions are the most studied integrin adhesion complexes (IACs), together with invadosomes. FAPs contain repeats of focal adhesion units arranged in parallel [2], whereas the invadosome is a cellular structure with an actin-rich core that can act like a “foot”, allowing cells to move [3]. Invadosomes include invadopodia and podosomes, which are essentially the same structure but are differentially termed according to their involvement in physiological (podosomes) or pathological (invadopodia) processes. In a tumor context, the invadosome plays a key role in the metastatic cascade [4].

FAPs mediate cell adhesion by connecting the cytoskeleton to the ECM, and together with matrix metalloproteinases (MMPs) also play an essential role in the migration of cells in both physiological and pathological processes. Accordingly, FAPs and MMPs have a role in cancer cell migration and invasion and are crucial for epithelial–mesenchymal transition (EMT) [5].

Many proteins are involved in FAPs, with a precise arrangement from the outside to the inside of the cells. ECM proteins and glycosaminoglycans (fibronectin, vimentin, elastin, collagen, hyaluronic acid) bind to integrins, which recruit intracellular anchoring proteins, such as talin and paxillin; these, in turn, bind other cytoplasmic proteins, such as focal adhesion kinases (FAK), whose phosphorylation state can modify cytoskeletal organization, with the participation of other molecules [6].

Due to their key role in cancer cell migration and invasion, MMPs and components of FAPs are often upregulated in cancer and are thus potential targets for cancer therapy.

Indeed, the inhibition of signaling pathways associated with focal adhesion dynamics or the disruption of the interactions between cancer cells and the ECM at focal adhesion sites are strategies under investigation for preventing or limiting metastasis. In this regard, several studies have assessed the effects of polyphenols on FAPs and MMPs. Polyphenols are natural compounds present in many dietary plants, with antioxidant, anti-inflammatory and anticancer properties [7,8].

In this review, we firstly describe the proteins involved in FAP structure and function, in particular integrins, signal transduction effectors and cytoskeleton proteins; next, we discuss the evidence obtained in in vitro and in vivo studies on the effects of polyphenols on FAP components and MMPs. Besides, a variety of studies show potential synergistic effects when polyphenol treatments are combined with chemotherapeutic drugs [9]. The ultimate aim is therefore to provide a better view and understanding of the potential possibilities offered by the future use of polyphenols as anti-cancer agents.

## 2. Focal Adhesion Plaques (FAPs): Structure and Functions

### 2.1. Integrins: Key Proteins in FAPs Formation

Integrins are a family of proteins which pair to form 24 types of heterodimers [10,11]. They all have a large extracellular portion of approximately 1600 amino acids and two smaller intracellular terminal tails of approximately 20–50 amino acids [10]. The heterodimers share a general structure consisting of two different chains, an α and a β chain. Eighteen α chains isoforms and 8 β chain isoforms have been identified, and through the different combinations, the 24 different heterodimers are created [11]. The different types of integrins can communicate with each other, as explained by Gonzales et al., who observed how the inhibition of one integrin can modify the binding affinity between a different integrin and its ligand, a phenomenon defined as “transdominant inhibition of integrin function”, in which talin appears to play a key role. The failure of this crosstalk seems to be implicated in some pathologies [12]. 

Integrins have a role in connecting and transmitting signals between the extracellular environment and the cytoskeleton. Moreover, in addition to providing an intracellular signal when they bind an extracellular ligand, integrins can also be regulated by signals generated inside the cell. Indeed, they can be activated through two different forms of signaling: inside-out and outside-in mechanisms. Inside-out signaling occurs when intracellular proteins, like talin and kindlin, bind to an inactive integrin cytoplasmic domain, leading to a conformational change of the extracellular domain, which results in integrin activation. The inside-out signaling is activated by a chemokine binding to its receptor and allows the recruitment of different proteins (vinculin, paxillin, FAK) to the intracellular portion of the integrin, triggering various signaling cascades involved in adhesion to the cytoskeleton, cell survival and migration processes. Moreover, the strong binding between active integrins and their ECM ligands initiates outside-in signaling by activating FAK and inducing Src-mediated phosphorylation of proteins within IACs and multiple downstream signaling pathways [13,14]. 

The association of focal adhesions with intracellular proteins, such as FAK/Src, has also been related with physiological and pathological processes, including tumor cell survival, progression and invasiveness [15]. Recent studies have also demonstrated how IACs, and proteins associated with them, are involved in the regulation of cell cycle and division [16].

In the tumor context, one mechanism by which integrins promote invasiveness is the activation of several genes involved in the EMT, which is marked by a switch between the expression of the epithelial cadherin (Epithelial cadherin, E-cadherin) and the mesenchymal isoform (Neural cadherin, N-cadherin) and by the expression of particular integrins such as the αV integrin [17]. Indeed, integrins and cadherins are inherently connected through the actin cytoskeleton and share signaling molecules. While the integrin–actin axis has been recognized for its ability to sense mechanical pressures, several studies showed that cadherins are also capable of mediating signal transduction and cells’ response. In fact, there is growing evidence that the actin cytoskeleton, intracellular forces, signaling intermediates of integrins and cadherins and their spatial distribution, are all regulated by mechanically driven crosstalk [18]. In addition to mesenchymal cadherins, different integrin complexes are known to increase their expression during EMT. These include α5β1, which binds to fibronectin, and α1β1 along with α2β1, that are connected with collagen I and have been demonstrated to cause the disruption of E-cadherin complexes [19].

During the EMT, cancer cells use developmental processes to acquire migratory and invasive properties that enable them to start the metastatic cascade and colonize different sites of the body. These events include a significant rearrangement of the actin cytoskeleton and the concurrent formation of membrane protrusions, such as lamellipodia, filopodia, invadopodia and podosomes, necessary for invasive growth [20].

### 2.2. Intracellular Proteins Involved in Signal Transduction and Their Scaffolding Role

Intracellular proteins involved in the formation, regulation and activity of FAPs are talin, kindlin, vinculin, paxillin and FAK. Talin binds with its Four-point-one, Ezrin, Radixin, Moesin (FERM) subdomain to the intracellular portion of the activated integrins (β3). This allows the recruitment of kindlin, and direct contact of the integrin with vinculin, which then binds the cytoskeleton (actin). FAK also binds talin, leading to FAK autophosphorylation and paxillin phosphorylation. Activated paxillin can thus bind to vinculin, thus stabilizing FAP structure and activating a signaling cascade for the transcription of genes involved in cells’ migration and survival [21]. 

Talin is a key element of FAPs because it is an important regulator of integrin activity [22,23,24]. Priddle et al. demonstrated the importance of talin in the formation of adhesion plaques by observing how in cells negative for the talin gene (−/−), FAP formation was completely impaired [25]. Talin also appears to be necessary for the recruitment of paxillin to the plaques, while it does not appear to be crucial for the adhesion-induced phosphorylation activation of FAK and Src, as demonstrated by Giannone et al. [26]. The recruitment of talin near the plaque is important in its initial formation, when the bond between integrins and the cytoskeleton is still weak, and when the cell undergoes mechanical stimuli that require the formation of even stronger bonds [26]. A recent study carried out by Di Paolo et al. demonstrated how talin exerts its action in the formation of FAPs through binding to phosphatidylinositol phosphate kinase type Iγ (PIPKIγ), leading to an increase in phosphatidylinositol(4,5)-bisphosphate (PIP2) close to the plaques and acting as feedback in the synthesis of other adhesion proteins such as vinculin and talin itself [27]. A specific isoform of PIPKIγ, PIPKIγ661, binds talin at the same site where talin binds the β tail of integrins; Src is able to phosphorylate PIPKIγ661 at residue Y644, increasing its affinity for talin, thus competing with integrins and regulating plaque formation [24].

Kindlins are a family of proteins involved in integrin activation. There are three main members of this family, kindlin-1 (expressed in epithelial tissues), kindlin-2 (expressed in many tissues) and kindlin-3 (expressed in hematopoietic cells). In the absence of kindlin, integrins are unable to switch from the low-affinity to the high-affinity state for their ligand [28]. Unlike talin, kindlins bind the distal NxxY motif on the β1, β2 and β3 tails of integrins. However, talin and kindlin are both necessary to correctly cluster integrins [29].

Vinculin is an essential component of the FAP, having the purpose of connecting the structures of the plaque itself with the proteins of the cytoskeleton [30]. It is composed of 1066 amino acids and has five distinct domains able to form bonds with other proteins such as paxillin, talin, actin and other vinculin molecules [31]. Vinculin, under normal conditions, is found in a closed conformation. When talin binds to integrins in response to stimuli, vinculin binds to talin, thus opening its structure and releasing the binding site for F-actin [32]. When the head of vinculin binds integrins, its tail forms bonds with the actin cytoskeleton, thus generating tensile forces that allow cells’ movement [32]. These forces are exerted by myosin bound to actin, which, being bound to vinculin, transmits them to the adhesion plaque, therefore generating movement [33]. It has been demonstrated that vinculin is necessary in maintaining adhesion to the substrate, as cells that overexpress vinculin have larger focal adhesions, while cells that express a smaller quantity of vinculin have a lower amount of focal adhesions [34]. Several studies suggest that vinculin contributes to maintaining the stability of adhesions thanks to the support of other proteins such as talin and paxillin [35,36]. Given the essential role of vinculin in the formation of FAPs, it is obvious that it is also involved in their destruction, a fundamental step in the process of cell migration. Some studies show how PIP2 and calpain may be involved in the inactivation of vinculin, thus leading to the disruption of focal adhesions. Specifically, PIP2 appears to be able to decrease the binding between vinculin and actin, as supported by the fact that cells in which PIP2 is overexpressed have fewer focal adhesions [37]. Calpain, a Ca^2+^-dependent protease, is instead capable of cleaving talin, breaking the bond with vinculin and disassembling the adhesions. In support of this theory, cells mutated in the calpain gene show an inhibition of adhesion plaques turnover [38].

One of the most important proteins of the FAP is FAK, a tyrosine kinase, which can bind to these structures thanks to a C-terminal region called FAT (Focal Adhesion Targeting). This binding domain is essential for FAK signaling activity, as demonstrated by Shen et al. [39]. The main action of FAK is to activate a series of signals and pathways involved in cell survival, tumorigenesis, invasiveness and cell mobility [40,41,42,43]. After the binding of integrins with their ligands, the activation of FAK occurs when its Y397 tyrosine residue is phosphorylated [42]. This modification leads to the recruitment of further kinases, such as Src, which in turn phosphorylates FAK in different tyrosine residues, such as Y576 and Y577, increasing its kinase activity [43]. However, Src is able to phosphorylate FAK also in different tyrosine residues, such as Y925, which is localized in the FAT region, leading to the detachment of FAK from the adhesion plaque [43]. All this evidence gives the idea of how the turnover of adhesion plaques is a fluid phenomenon and also how FAK is an extremely crucial protein in its regulation, as further demonstrated by Ilic et al., who observed that in FAK-deficient mice (−/−), there was an increase in FAP formation and a decrease in cell migration [44]. 

Lastly, paxillin plays a very important role as a scaffold protein in focal adhesions. It has several binding domains including a proline-rich motif able to bind the SH3 domain of Src and five LD motifs able to bind several proteins including FAK [45] and can also directly bind the cytoplasmic β1 and α4 tails of integrins [46]. The tyrosine residues that cause paxillin activation are Y31 and Y118, which can bind various proteins such as Crk [47]. This binding is essential to allow paxillin localization in the adhesion plaque and carry out its effect in cell migration [48], which can be positive or negative depending on the cell type and the type of binding with Crk [49,50,51]. Binding to the α4 subunits of integrins leads to an inhibition of α4β1-dependent cell migration [51]. Paxillin can also activate small GTPases, such as Rac, through the formation of a protein complex with β-PIX and GIT2/PKL, triggering a signaling cascade that leads to an increase in cell migration [48], again confirming the complexity of FAP structure.

## 3. Matrix Metalloproteinases and Their Role in the Invasion Process

MMPs are a family of Zn-dependent metalloenzymes with the characteristic ability to degrade ECM proteins [52]. They are divided into three subfamilies depending on the substrate they recognize: interstitial collagenases, stromelysins and gelatinases [52]. The overall activity of MMPs is regulated at all levels, from transcription to post-translational modifications [52].

In most cases, the transcription of the genes coding for MMPs is regulated by hormones and growth factors such as transforming growth factor-alpha (TGF-α), platelet-derived growth factor (PDGF), epidermal growth factor (EGF), interleukin-1β (IL-1β), nerve growth factor (NGF), tumor necrosis factor-α (TNF-α), with IL-1α appearing to be the activator required for the synthesis of interstitial collagenases [53]. MMPs’ expression is also stimulated by certain conditions such as calcium influx [54], UV light exposure [55], and cell shape changes [56]. MMPs’ activation process has now been elucidated and consists of the so-called “cysteine switch”: a cysteine residue within the site where the Zn atom is located forms a bond with it and maintains the enzyme in latent form [57]; when this bond is interrupted for physical and/or chemical reasons, a conformational change occurs followed by a proteolytic cleavage which activates the enzyme. One of the ways in which cysteine–Zn binding is disrupted appears to involve the plasmin activation cascade [58]. Logically, there is a way to regulate the action of MMPs so as to maintain their physiological role and prevent their pathological action, and this regulation is carried out by the family of tissue inhibitors of MMPs (TIMPs). Three different isoforms of TIMP, TIMP-1, TIMP-2 and TIMP-3 have been isolated [59]. TIMP-1 is a glycoprotein capable of forming a 1:1 bond with collagenases even in their latent form, inhibiting their activation and catalytic activity [60]. TIMP-2 is a non-glycosylated protein and is highly specific for gelatinase A, forming a 1:1 bond with it even in the latent form and inhibiting its proteolytic activity and activation [61]. Finally, TIMP-3, unlike the others that are secreted by the cell and remain soluble in the ECM, is already localized in the ECM and tends to form stable bonds with its components [62].

The majority of malignant tumors show an overexpression of these endopeptidases, which allow them to have a greater ability to infiltrate surrounding tissues and form metastases. The MMPs mainly involved in tumor invasion and present in invadopodia are certainly MMP-9 and MMP-2. These two MMPs have the specific ability to degrade type IV collagen present in the basement membrane, allowing tumor cells to invade. This degradation also exposes new portions of type IV collagen which can be recognized by the integrins expressed on the surface of the tumor cells, causing their activation and the ensuing cell survival and migration processes [63].

## 4. Polyphenols

**Figure 1:**
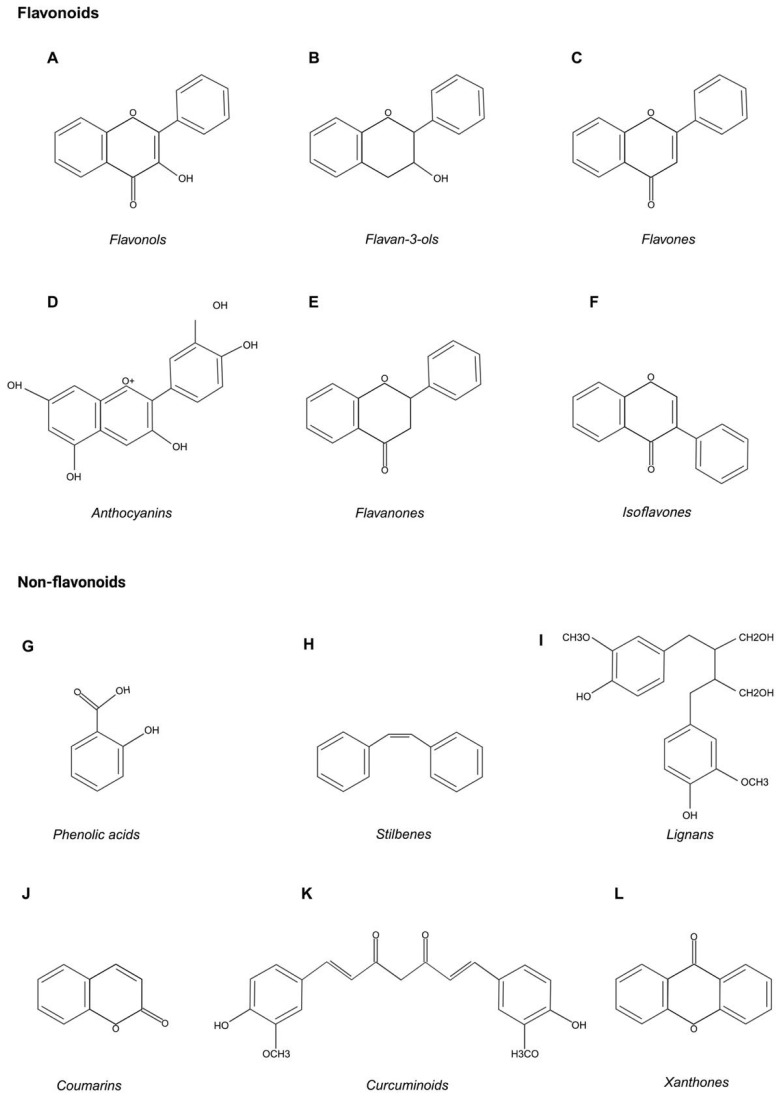
Polyphenols classification. The figure was created with BioRender.com (accessed on 12 January 2024).

Polyphenols are a large group of natural compounds derived from plants and consequently found in beverages and food such as fruits, vegetables, spices, cereals, nuts, legumes, olives, tea, coffee and wine [64]. Polyphenols can be classified into flavonoids and non-flavonoids based on their chemical structures (Figure 1) [65]. 

A plethora of studies indicate that natural polyphenols may be used in both cancer prevention and treatment, thanks to their antioxidant and anti-inflammatory activities, as well as to the modulation of multiple molecular events involved in carcinogenesis [66]. Due to their ability to simultaneously interact with several pathways implicated in carcinogenesis, polyphenols can prevent the proliferation of cancer cells [67]. Both in vitro and in vivo, polyphenols have the ability to modulate several signal transduction pathways involved in the genesis of different cancers. For instance, these compounds have shown strong effects on transcription factors such as nuclear factor erythroid 2 (Nrf2), β−catenin, peroxisome proliferator activator receptor-gamma (PPAR-γ), signal transducer and activator of transcription 3 (STAT3), nuclear factor-κB (NF-κB) and activator protein-1 (AP-1). Polyphenols have also been shown to target growth factor receptors [epidermal growth factor receptor (EGFR), ErbB2, vascular endothelial growth factor (VEGFR) and Insulin-like growth factor 1 receptor (IGF1-R)], protein kinases [(RAS/RAF, mammalian target of rapamycin (mTOR), phosphatidylinositol 3-kinase (PI3K), BCR-ABL and AMP-activated protein kinase (AMPK)] and pro-inflammatory mediators [interleukins (ILs), tumor necrosis factor-α (TNF-α), cyclooxygenase-2 (COX-2), and 5-lipoxygenase (5-LOX)]. Furthermore, polyphenols have both pro- and anti-oxidant characteristics [68]. For instance, neoplastic transformation is significantly influenced by the interaction of signaling pathways regulated by ErbB receptors, NF-κB and the Hedgehog (Hh)/glioma-associated (GLI) oncogene cascade (Hh/GLI). Indeed, the Hh signaling cascade ends through the actions of GLI zinc finger transcription factors, which regulate gene expression, and it has been observed that the EGFR and the Hh/GLI pathways work together to synergically cause oncogenic transformation, which largely depends on EGFR-mediated activation of the RAS/RAF/MEK/ERK pathway [69,70]. In addition, it has also been observed that inhibitory-κB kinase α (IKKα) increases the invasive potential of ErbB2-positive breast cancer cells, which in turn causes ErbB2 to activate NF-κB via the canonical pathway [71]. Moreover, cell transformation can result from deregulation of MAPK; indeed, 40% of malignant tumors have mutations in the RAS–RAF–MEK–ERK pathway, mostly in RAS. Due to their ability to modulate multiple signaling pathways, including PI3K/Akt and MAPK, as well as key proteins involved in the development of cancer, such as p53 and RAS, polyphenols are ideal candidates with potential therapeutic effects for the prevention or treatment of various types of cancer. For example, by downregulating phospho-ERK in pancreatic cancer cells, resveratrol inhibits the MAPK signaling pathway and nicotine’s potential to stimulate cell growth; meanwhile, kaempferol is able to inhibit the activity of MMP-9, by deactivating the MAPK/AP-1 pathway in breast cancer cells [72]. It has also been reported how polyphenols can interact with neural receptors and signaling pathways, for example the PI3K/Akt and ERK1/2 pathways, thereby influencing cellular responses [73,74,75,76]. Specifically, by modulating different cellular functions, polyphenols play an important role in neuroprotection being antioxidant, anti-inflammatory and anti-apoptotic. These functions are carried out by activating pathways such as the PI3K/Akt, Akt-ERK1/2 and MAPK [77]. Moreover, polyphenols have also been found to interact with neural substrates involved in the modulation of cytoskeletal dynamics [73,74,75,76]. 

Polyphenols also have the ability to influence several processes involved in carcinogenesis, including cell cycle, apoptosis, angiogenesis and autophagy. For example, in normal cells, autophagy has an anticarcinogenic effect and prevents cells from malignant transformation. Conversely, anomalies in autophagy are linked to drug resistance, invasion and metastasis [78]. Emerging evidence suggests that polyphenols promote protective autophagy, but they can also cause autophagic cell death in cancer cells. In this regard, several studies have shown how polyphenols are able to induce cytoprotective autophagy by promoting the autophagosome formation via the conversion of LC3-I to LC3-II, decreasing PI3K/Akt/mTOR and Akt/STAT3 pathways and increasing proteins such as Beclin 1, ATG5, ATG7, ERK, AMPK and ULK1 [65]. During carcinogenesis, loss of function mutations in tumor suppressor genes, such as *p53*, can prevent apoptosis, promote cell survival and the development of cancer. In this context, several effects have been ascribed to polyphenols, including the upregulation of the pro-apoptotic cleaved caspases-3, -8, and -9 and PARP and the downregulation of the anti-apoptotic Bcl-2 and Bcl-xL, leading to the loss of mitochondrial membrane potential and the release of cytochrome c, which all contribute to the decrease in cell proliferation, cell cycle arrest and induction of cell death [79]. Reactive oxygen species (ROS) have multiple impacts on the development and incidence of cancer, which is why antioxidants have drawn attention as potential therapeutics. Flavonoids are naturally occurring polyphenols that have been shown to have anticancer and antioxidant properties. Free radicals (R-O•) are reduced by flavonoids by receiving an electron and a hydrogen atom from them. Moreover, flavonoids prevent nuclear NF-κB response element (NF-κB RE) from binding to NF-κB and operate as ligands to activate PPARγ, which in turn suppresses the NF-κB RE. The expression of COX-2 is attributed to this response element. Consequently, reduced oxidative stress is the outcome of flavonoids’ dual inhibition of COX-2 transcription. Lastly, flavonoids prevent Keap1 from binding itself to Nrf2, and this promotes the production of phase II detoxifying enzymes by enabling Nrf2 nuclear translocation and activation of electrophile response element (EpRE) [80]. Because of all these considerations, polyphenols are potential drugs for cancer therapies, even though further investigations are required to fully understand the mechanisms underlying polyphenol-induced regulation of cancer. 

### 4.1. Flavonoids: Classification and Description 

Flavonoids originate from phenylalanine and are well present in the daily food intake [81]. The chemical structure includes 15 carbon atoms with two aromatic rings (A, B) linked by a three-carbon bridge, composing a heterocyclic ring (ring C), hence C6–C3–C6 [82,83]. Flavonoids are classified into subclasses, according to the different functional groups, ring C’ level of oxidation and possible combinations of rings B and C [84]. The main subclasses are flavonols, flavones, flavan-3-ols, anthocyanins, flavanones and isoflavones [65,83]. The primary flavonoid core can have numerous substituents. Hydroxyl groups can be found at positions 40-, 5- and 7-. Those are very common as most flavonoids exist as glycosides and have increased water solubility; instead, other groups generate lipophilic flavonoids, like methyl groups and isopentyl units [83].

Flavonols are formed by an unsaturated C ring at C2-C3, hydroxylated at C3 and oxidized at C4 (Figure 1A) [85]. The main flavonols are quercetin, kaempferol, myricetin, rutin and fisetin [65]. They can mainly be found in tea, red wine, fruits and vegetables and are identified as the most common and largest flavonoid subgroup. Resembling flavones, flavonols differ in methylation and hydroxylation forms, specifically hydroxyl groups play a major role in biological activities, especially in antioxidant activity [85].

Flavan-3-ols originate in flavans, which have a 2-phenyl-3,4-dihydro-2H-chromen-3-ol skeleton (Figure 1B) [86]. They can display a variety of structures and consist of a series of compounds, such as catechin, epicatechin, epigallocatechin, gallocatechin and proanthocyanidins [65]. Flavan-3-ols contribute to plant defense and are found in most plants [87]. These compounds also show several health beneficial properties like antioxidant, cardiopreventive, anticarcinogen, antiviral, antimicrobial and neuroprotective properties [86].

Flavones have a double bond between C2 and C3 in the flavonoid skeleton and are oxidized at C4 (Figure 1C) [88]. The synthesis of flavones in parsley cells can be upregulated by UV light, leading to a production of apigenin and luteolin in concentrations >20 times higher in celery leaves than in stalks [89]. Flavones also have important antioxidant properties thanks to their ability to scavenge ROS [90]. Indeed, luteolin was found to be one of the most potent inhibitors of xanthine oxidase, a key enzyme in ROS production. Flavones also have anti-inflammatory and anticancer functions [91].

A class of water-soluble flavonoids found in abundance in fruits and vegetables are called anthocyanins. Berries (both red and purple), grapes, apples, plums, cabbage and foods with high natural colorant content are dietary sources of anthocyanins. The six common anthocyanins are pelargonidin, peonidin, petunidin, delphinidin and cyanidin (Figure 1D). These substances are vacuolar pigments that dissolve in water, primarily found in fruits and flowers but also found in vegetative organs [92,93,94]. The production of anthocyanins is stimulated by unfavorable circumstances including biotic and abiotic stressors [95]. Numerous in vitro studies have assessed the biological and pharmacological potential of these molecules and shown that they have the ability to function as antimicrobial agents, counteract oxidative stress and prevent the onset and progression of a variety of non-communicable diseases, including cancer, heart disease, neurological and metabolic disorders [95]. Together with other polyphenols and flavonoids, anthocyanins have the capacity to function as free radical scavengers against hazardous oxidants such as reactive nitrogen species (RNS) [96,97,98]. 

Flavanones are natural compounds that have a variety of aromatic components and substantial activity (Figure 1E). Flavanones show great promise in the treatment of cardiovascular disorders, cancer and many other conditions. The chemical alteration of flavanones determines their bioavailability and bioactivity. There are two categories of flavanones: naturally occurring and artificially produced. A broad spectrum of compounds with A- or B-ring substitutions, such as hydroxy, methoxy, methylenedioxy, O- and C-glycosyl, C-methyl, C-benzyl, C-hydroxymethyl, C-formyl and C-isoprenyl substituents (including furano or dihydrofurano rings), conjugations to stilbene, anastatin, phenolic acid and diarylheptanoid moieties, are included in the flavanone class. Flavanones are found in nature in both aglycone and glycosidic forms in every section of numerous higher plant families, including the Rutaceae, Compositae and Leguminosae [99]. However, citrus fruits including grapefruit, sweet and sour oranges and tangelos have the highest concentrations of these chemicals [99]. Because of their great frequency in food, the flavanones naringenin and hesperetin in the forms of aglycone and glycoside are of particular interest. Naringenin is a hydroxyl derivative of flavanone (5,7,4′-trihydroxyflavanone) and has anti-inflammatory, anti-ulcer, diastolic, estrogenic and skin-protective properties [100,101,102]. Hesperetin (4′-methoxy-5,7,3′-trihydroxyflavanone) is the primary flavonoid in lemons, limes, oranges, tangerines and tangor species of citrus fruits, where it is found as glycoside (hesperidin). Like naringerin, hesperetin is a flavanone that lowers cholesterol but also has hypolipidemic, anticoagulant, antioxidant, anticancer, anti-inflammatory, antiviral and antifungal properties. Because of its characteristics, hesperetin might be useful in the treatment of hypertension [99,103,104,105]. According to recent research, flavonoids may have advantageous neuropharmacological effects, such as anticonvulsant and antidepressant qualities, and hesperetin may be able to shield neurons from oxidative or nitrosative stress-related damage. Through mechanisms that differ from those of traditional antidepressants, citrus flavanones also exhibit antidepressant effects [104]. In addition, hesperetin inhibits the intracellular reproduction of certain viruses. One more flavanone compound is 2′-hydroxyflavanone, that was extracted from the complete plant of *Mimosa pudica* (L.) and reported to have anti-inflammatory properties in vitro [106]. 

Isoflavones are phytoestrogens, i.e., low-molecular substances that plants produce and store in response to stress and microbial attacks (Figure 1F). In plants, they serve as phytoalexins rather than hormones. These molecules, which are active defense factors, possess antioxidant, antiviral, fungistatic and antibacterial qualities [107,108,109]. Legumes from the family Fabaceae are the primary source of isoflavones [110]. Specifically, red clover (*Trifolium pratense*) contains formononetin and biochanin A, while soybeans (*Glycine max*) contain daidzein, genistein and glycitein. Genistein (7,4′-dihydroxy-6-methoxyisoflavone), daidzein (7,4′-dihydroxyisoflavone), glycitein (7,4′-dihydroxy-6-methoxyisoflavone), biochanin A (5,7-dihydroxy-4′-methoxyisoflavone) and formononetin (7-hydroxy-4′-methoxyisoflavone) are isoflavone phytoestrogens [111]. Isoflavones can exist as glycosides or aglycons and can generate 6-*O*-malonylglycosides, 6-*O*-acetylglycosides, 7-*O* or, in some plant species, 8-C-β-d-glycosides [112]. As an alternative treatment for a variety of hormonal-related disorders, such as breast and prostate cancers [109,113], cardiovascular diseases [114], osteoporosis [115] or menopausal symptoms [116,117], isoflavones are thought to be chemoprotective [118]. Isoflavones might also be regarded as endocrine disruptors, potentially having detrimental effects on the environment [119] or on the health of a specific population segment [120,121]. Despite having a distinct chemical structure from endogenous estrogens, isoflavones share a phenol group that allows them to connect to and activate estrogen receptors (ER) [122]. Isoflavones bind to ER in target tissue cell to control gene expression. They do not, however, stay in the cell nucleus for very long. Phytoestrogens act as estrogen agonists or antagonists: when they bind to the ER, they can hinder the actions of endogenous estrogens, whereas they function as weak estrogens when there is a condition of estrogen deficiency [123]. 

### 4.2. Non-Flavonoids: Classification and Description

Although the structural skeleton of polyphenols contains several hydroxyl groups on aromatic rings, the basic structure of non-flavonoids is a single aromatic ring (Figure 1). Phenolic acids, stilbenes, lignans, coumarins, curcuminoids and xanthones are examples of non-flavonoid compounds [65]. 

Phenolic acids, principally their derivatives from benzoic and cinnamic acids, constitute the main class of this group [124]. Benzoic acid and cinnamic acid are chemical precursors of phenolic acids. Few edible plants contain hydroxybenzoic acids, which have a C6-C1 structure and low nutritional value (Figure 1G). Gallic acid (GA), the most common phenolic acid, and protocatechuic acid are members of this subclass. Grapes, wine, green and black teas and mangoes are the primary dietary sources of GA in its non-sugar galloyl ester form. Additionally, GA is present as complex sugar esters and is the biosynthetic precursor of hydrolysable tannins (gallotannins and ellagitannins) [83]. Hydroxycinnamic acids with a C6–C3 structure are mostly found as glycosylated forms or as tartaric, quinic and shikimic acid esters: caffeic acid, ferulic acid, p-coumaric acid and sinapic acid are the most prevalent hydroxycinnamic acids. The most common phenolic acid, caffeine, makes up 75–100% of the total hydroxycinnamic acid in most fruits [125]. The biological actions of phenolic acids, including their anti-inflammatory, anti-atherosclerotic, immunoregulatory, anti-allergenic, anti-thrombolytic, antimicrobial, antitumor, anti-obesity, anticancer and anti-diabetic qualities, have generated increased interest [126].

Stilbenes are produced by plants, particularly berries, peanuts, rhubarb, grapes and others, as means of self-defense in stressful environments caused by pathogens and UV radiation [127]. Stilbenes are specialized metabolites formed by a C6–C2–C6 (1,2-diphenylethylene) structure (Figure 1H) [128]. Stilbenes are synthetized in a small but diverse group of plants because stilbene synthase (STS) is rarely present [127]. Stilbenes include resveratrol and pterostilbene: these compounds have many interesting health-promoting properties, such as anticancer, anti-inflammatory, anti-aging, antioxidant and anti-atherogenic effects [128,129].

Lignans, which are secondary metabolites found in vascular plants, are widely distributed throughout the plant kingdom and are linked to a variety of physiological processes that are beneficial to human health. They originate from the biosynthetic pathway of shikimic acid. These secondary plant metabolites are classified as diphenolic compounds as they are derived from the combination of two phenylpropanoid C6–C3 units at the β and β′ carbon (Figure 1I). Their chemical structure is similar to the 1,4-diarylbutan, and they can be attached to additional ether, lactone or carbon bonds [130]. The most commonly studied compounds are pinoresinol, lariciresinol, arctigenin, honokiol, sesamin, magnolol, seicolariciresinol, matairesinol and medioresinol [65]. In the kingdom of plants, they have been found in about 70 families, including grains, vegetables, trees and grasses: roots, rhizomes, stems, leaves, seeds and fruits all contain lignans [131]. Several biological activities of lignans have been suggested, including anticancerous, antioxidant [132], antiviral [133], antibacterial [134] and immunosuppressive properties [135].

Coumarin and its derivates are alpha-benzopyrones and can be classified into four main categories: (i) simple coumarins, (ii) furano coumarins, (iii) pyrano coumarins and (iv) dicoumarins. Coumarins (1,2-benzopyrone or 2H-1-benzopyran-2-one) comprise a very large class of compounds that can be found in fruits (at high levels), leaves, flowers, stems and roots of several plants, especially Rutaceae and Apiaceae [65]. Coumarin, which is present in cinnamon and other plants, is the prototype of this class of molecules (Figure 1J) [136]. Coumarin and its derivates have gained significance in recent times: investigations into the biological activity of coumarin derivatives have demonstrated that these compounds exhibit a large range of biological activities, including antitumor, antibacterial, antifungal, anti-inflammatory, anticoagulant (vitamin K epoxide reductase) and triglyceride-lowering effects. Natural sources are notable in this context because of their low toxicity, low drug resistance, low cost and good efficacy. Moreover, new compounds extracted from natural sources and the combination of these substances with established chemotherapeutic drugs could represent future therapeutic strategies to improve patient outcomes, especially in cancer patients [137,138].

Curcuminoids are diketone compounds that include curcumin, demethoxycurcumin and bisdemethoxycurcumin, each of which has a unique substituent for the benzene ring. The main member of the curcuminoids subclass is curcumin (1,7-bis-(4-hydroxy-3-methoxyphenyl)-1,6-heptadiene-3,5-dione). Curcumin, which is widely used in cooking, was first discovered in 1910, derives from the rhizome of the plant *Curcuma longa* and is found in the spice turmeric [139]. This compound has several functional groups where the planar aromatic ring systems are attached to α,β-unsaturated carbonyl groups (Figure 1K). Since curcumin can alter several targets and signaling pathways connected to cancer, it is regarded as a pleiotropic molecule and a multifunctional drug [84]. Both synthetic and natural curcuminoids have a variety of biological activities, including anti-inflammatory [139], antioxidant [140], anticancer [141,142], antimicrobial [143] and anti-Alzheimer’s disease properties [144].

Xanthones are a significant group of oxygenated heterocycles that are found in fungi, higher plants and lichens [145] as secondary metabolites. The core tricyclic skeleton of xanthones consists of one pyranoid ring and two benzenoid rings (Figure 1L) [146]. Three major families and seven major genera of higher plants, the Anacardiaceae (*Mangifera*), Gentianaceae (*Gentiana* and *Swertia*) and Guttiferae (*Calophyllum*, *Garcinia*, *Platonia* and *Hypericum*), have been reported to be rich in xanthones [147]. The biological activities of xanthones are extensively described, with a focus on how they might be used as therapeutic agents. Biological and pharmacological activities of natural xanthones comprise antimicrobial, antiviral, antioxidant, anti-atherosclerotic and cardioprotective effects [148,149]. The most prevalent xanthone isolated from the mangosteen, α-mangostin, received a lot of interest due to its anticancer effects observed in numerous studies on cancer cell lines and animal models [150]. While the mechanisms underlying these anticancer effects have been extensively studied, those of minor xanthones, such as gartanin, β-mangostin, γ-mangostin, garcinone C and garcinone E, as well as those of extracts from the pericarp, roots, rind and stem of mangosteens, are less understood and still need to be investigated [151].

## 5. Effects of Polyphenols on FAPs and MMPs

**Figure 2:**
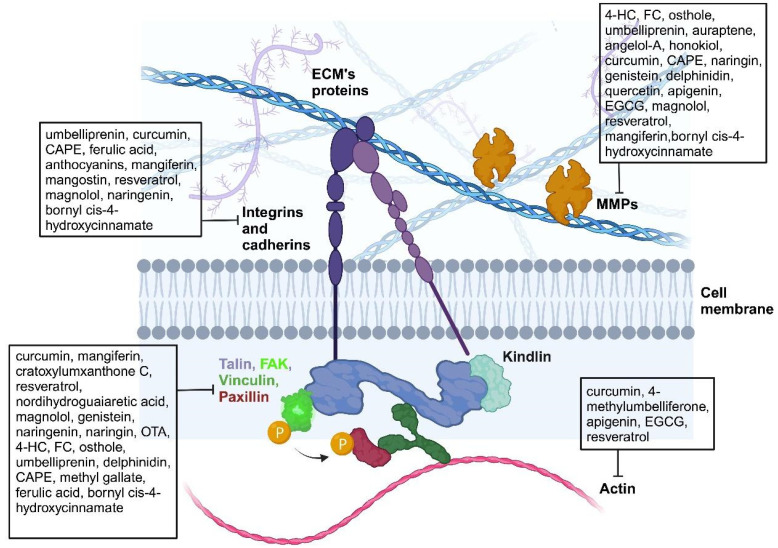
Representation of the effects of polyphenols on Focal Adhesion Plaques (FAPs) components and MMPs. MMPs, integrins and intracellular proteins cited in the text (Talin, Kindlin, Vinculin, Paxillin, FAK) are reported in the figure. The inhibition arc indicates a negative activity on FAPs’ components and MMPs exerted by the polyphenols reported in the boxes and described in the text. The figure was created with BioRender.com (accessed on 12 January 2024). Abbreviations: 4-HC, 4-Hydroxycoumarin; CAPE, Caffeic acid phenethyl ester; ECM, extracellular matrix; EGCG, Epigallocatechin-3-gallate; FC, Farnesiferol C; MMPs, matrix metalloproteinases; OTA, Ochratoxin A.

Several in vitro and in vivo studies investigated the effects of the different classes of polyphenols on FAP components and MMPs. In particular, as reported in the next sections, polyphenols are able to affect the functions of integrins and cadherins, MMPs, cytoskeleton components and intracellular proteins like FAK, paxillin, vimentin and talin (Figure 2). The effect of polyphenols on MMPs’ synthesis and expression has been frequently evaluated by Western blotting, while their enzymatic activity was assessed by wound healing assay. As for the formation and development of metastases, these have been observed using immunohistochemical techniques.

### 5.1. Effects of Polyphenols on Integrins and Cadherins

Through the cytoskeleton, integrins and cadherins are tightly connected to each other and, when deregulated, can promote migration and invasion of tumor cells through various mechanisms, including the EMT. Although cadherins are not directly part of the FAPs, numerous studies have shown how the action of specific polyphenols can revert the EMT, for example, by promoting the switch from mesenchymal cadherin (N-cadherin) to its epithelial isoform (E-cadherin) [17].

For the differentiation and function of epithelial cells, both cell–matrix and cell–cell adhesions are critical. In addition to mediating cell-to-cell interactions, classical cadherins are strong activators of signal transducer and activator of transcription 3 (STAT3), providing survival signaling. (E)-cadherin is necessary for cells to stay closely associated within differentiated epithelial tissues. The FAK/Src complex is bound by integrin receptors, which mediate cell adhesion to the ECM. Consequently, coordinated activation of the complementary cadherin/STAT3 and integrin/FAK pathways can significantly improve the survival and growth of tumor cells [152]. 

This section reports how different polyphenols interact with integrins and cadherins, resulting in the reduction in motility and proliferation of cancer cells.

Tumor progression and metastasis are significantly influenced by the overexpression of N-cadherin. Since p130CAS enables FAK to upregulate N-cadherin expression in pancreatic cancer cells, Vu et al. wondered if the flavan-3-ol epigallocatechin-3-gallate (EGCG) could inhibit FAK and reduce N-cadherin expression. In fact, they reported that N-cadherin expression is abolished in BxPC-3 pancreatic cancer cells treated with EGCG, which is consistent with the inhibition of FAK activity [153]. In another study, Sen et al. investigated the effect of EGCG on signaling molecules that may be involved in the control of MMP-2 activation in the human breast cancer cell line MCF-7. They observed that the adherence of MCF-7 cells to ECM, vitronectin and fibronectin was decreased by EGCG treatment. EGCG (>20 µM) resulted in reduced levels of integrin receptors α5, β1, αV and β3 mRNAs [154].

Flavones, such as luteolin and apigenin, were found to reduce the expression of adhesion molecules in different studies. In particular, luteolin (0.50 µM) considerably reduced the expression of adhesion molecules on the surface of human umbilical vein endothelial cells (HUVECs) and inhibited TNF-α-induced ICAM-1 expression [155]. Hasnat et al. evaluated the effect of apigenin on the expression of integrin subunits 4, 5, V and 3 in melanoma cells. Integrin subunits 4, 5, V and 3 were clearly downregulated in melanoma cell lysates after apigenin treatment (15 µM) [156].

Through their interaction with β-catenin, cadherins regulate cytoskeleton remodeling and adhesion junctions, stabilizing adhesive contact and polarization of epithelial cells [157]. Zhang et al. employed the adenomatous polyposis coli multiple intestinal neoplasia (Apc^Min/+^) mouse model to evaluate the effects of naringin on intestinal tumorigenesis. Naringin suppressed the expression of β-catenin and regulated GSK-3β activity in intestinal adenomatous cells, blocking tumorigenesis progression [158]. In another study, Han et al. demonstrated that naringenin downregulates the expression of mesenchymal-like markers and upregulates E-cadherin, which all contribute to a reduction in PC-3 prostate cancer cell migration [159]. A significant increase in the E-cadherin marker was also observed in vitro and in vivo in colorectal cancer (CRC) cells upon treatment with umbelliprenin, a sesquiterpene coumarin. It was suggested that the umbelliprenin (>100 µM)-induced increase in E-cadherin expression could result in stable cell connections and likely prevent the expression of beta-cadherin-induced metastatic factors [160]. Similarly, curcumin dose-dependently upregulated the expression of E-cadherin in CRC cells. Consequently, curcumin enhanced intact or tight cell–cell connections and reduced EMT, preventing CRC cell invasion, migration and metastasis [161]. Mani et al. demonstrated that tumor cell adhesion and chemotaxis are significantly reduced by curcumin plus light on bladder carcinoma and HUVEC cells. In particular, curcumin combined with exposure to visible light reduced all integrin subtypes expressed on these cells (α2, α3, α5, α6, β1 and β3) [162].

Yang et al. showed that bornyl cis-4-hydroxycinnamate, a phenolic acid isolated from *Piper betle* stems (20 µM), significantly and dose-dependently inhibits the EMT and thus the migration of A2058 and A375 melanoma cells, by increasing E-cadherin protein levels and by decreasing Snail and N-cadherin protein levels [163]. Similarly, Yu et al. demonstrated that E-cadherin expression significantly increases in human head and neck squamous cell carcinoma (HNSCC) cells upon caffeic acid phenethyl ester (CAPE) treatment. These findings suggested that CAPE (>50 µM), at least in part, could enable the modification of EMT status through the induction of E-cadherin expression, thereby limiting the aggressive behavior of HNSCC cells in vitro [164]. The increase in E-cadherin expression, along with the inhibition of MMP-2/MMP-9 expression and thus of EMT, was also observed upon α-mangostin treatment in pancreatic cancer cell lines MIAPaCa-3 and BxPCa-2 [165]. Chei et al. observed that magnolol downregulates N-cadherin and upregulates E-cadherin, effectively reducing EMT in human CRC cells HCT116 and SW480. Moreover, in these cell lines, magnolol was able to inhibit both TGF-β-induced cell invasion and EMT [166]. Cheng et al. demonstrated that magnolol inhibits the migration of LN229 and U87MG glioma cells and reduces the expression of proteins linked to focal adhesions. In particular, N-cadherin and β-catenin showed decreased membrane levels in response to magnolol. In addition, the presence of magnolol enhanced the interaction between N-cadherin and β-catenin. Moreover, in an orthotropic xenograft model, magnolol treatment (5 µM) decreased the expression of N-cadherin and phospho-MLC proteins, in addition to inhibiting tumor progression [167]. Similarly, mangiferin has been demonstrated to strongly inhibit β-catenin pathway’s activation in breast cancer cell lines in vitro and in vivo [168]. Indeed, upon mangiferin treatment, it was observed a higher expression of E-cadherin and a lower expression of MMP-7, MMP-9, vimentin and active β-catenin [168,169].

Brockmueller et al. investigated the involvement of β1-integrin receptors in the anti-invasive and antimetastatic properties of the stilbene resveratrol employing two CRC cell lines (HCT116, RKO). Their findings demonstrated that resveratrol dose-dependently inhibits the adhesion adapter protein paxillin, which promotes migration, concurrently upregulating the expression of E-cadherin. Resveratrol (2.5 µM) also prevented tumor microenvironment-induced p65-NF-κB phosphorylation and nuclear translocation, which is linked to modifications in the expression patterns of EMT biomarkers (slug, vimentin, E-cadherin), factors related to metastasis (CXCR4, MMP-9, FAK) and apoptosis (caspase-3) [170]. In addition, Buhrmann et al. examined the molecular mechanisms underlying resveratrol’s effects on TNF-β/TNF-βR-induced EMT and migration in CRC cells (HCT116, RKO, SW480). Similar to TNF-α, TNF-β caused a significant increase in cell proliferation and morphological changes in CRC cells, which included the formation of filopodia and lamellipodia, the acquisition of an epithelial-like mesenchymal shape, a reduction in E-cadherin expression and increased migration/invasion. The treatment of CRC cells with resveratrol induced an increase in the expression of E-cadherin and a clear inhibition of vimentin expression. On the whole, these findings indicated that after TNF-β treatment, all three CRC cell lines underwent EMT and that resveratrol (5 µM) was able to prevent this induction [171].

Important steps in the multistep cascade of migration and metastasis involve the expression of receptors on endothelial cells and CAMs on tumor cells. Mostafa et al. discovered that several phenolic acids and other metabolites produced by the fermentation of anthocyanins by microbiota can inhibit the phosphorylation of signaling proteins, a process that consequently inhibits the migration of cancer cells. They tested plasma anthocyanins and their metabolites (>100 μM) on two pancreatic cancer cell lines (PANC-1 and AsPC-1) and showed that the plasma metabolites extracted after juice intake significantly reduced the expression of β1-integrin and ICAM-1 on PANC-1 cells [172].

In conclusion, the reported findings demonstrate that polyphenols are able to inhibit the enzymatic activity of integrins and in some cases to favor the switch from the mesenchymal to the epithelial form of integrins and cadherins, thus reversing the EMT (Table 1).

### 5.2. Effects of Polyphenols on FAP Intracellular Proteins

The ultimate effect of signal transmission from the ECM to the inside of the cell depends on the action of intracellular proteins, such as FAK, ERK, Akt, able to bind membrane receptors, such as integrins, and translate the input signal via the activation of factors, such as NF-κB and AP-1, capable of increasing the transcription levels of genes involved in cell survival and migration. 

Flavonols were shown to affect FAK phosphorylation [173,174] and to inhibit the STAT3 signaling pathway, IL-6-induced STAT3 signaling, IL-6-induced EMT [175], as well as PI3K/Akt [174] and ERK1/2-pathways [176]. In detail, Lee et al. found that quercetin (>50 µM) greatly reduces FAK phosphorylation and suppresses the invasive potential and migration of pancreatic cancer and epidermoid carcinoma cells in vitro [173]. Further, quercetin (>100 µM) reduced FAK, ERK1/2 and phospho (p)-ERK1/2 levels, inhibiting the migration and invasion of SAS human oral squamous cell carcinoma cells [176]. Similarly, Hung et al. demonstrated that kaempferol (50 µM) reduces FAK phosphorylation but also inhibits the PI3K/Akt pathway, resulting in suppression of cell invasion, migration and metastasis in human renal cell adenocarcinoma (786-O) and human kidney-2 (HK-2) cells [174]. In addition, it has been reported that quercetin (>50 µM) reverses IL-6-induced EMT, invasion and migration of PC cancer cells by blocking the STAT3 signaling pathway [175]. Quercetin treatment (80 µM) also suppressed the phosphorylation of c-Met and its downstream effectors including Gab1 (GRB2-associated-binding protein 1), FAK and PAK (p21-activated kinases) in the human medulloblastoma cell line DAOY, human hepatoma HepG2 and melanoma A375 and A2058 cell lines [177]. Like flavonols, flavan-3-ols (50 µM) are found to have a potential use in cancer treatment considering that they can inhibit FAK and significantly lower the levels of phospho-ERK (20 µM), as demonstrated in pancreatic cancer, breast cancer, melanoma and fibrosarcoma cells [153,154]. The flavone luteolin was reported to block EGFR tyrosine kinase activity, which resulted in the reduction in FAK expression also accompanied by lower FAK tyrosine phosphorylation levels, leading to cell invasion inhibition in MiaPaCa-2 and A431 tumor cell lines [173]. Moreover, studies performed in different cancer cell lines showed that apigenin is able to inhibit FAK expression [178], FAK/Src activation, which affected motility and cytoskeleton remodeling (25 µM) [179], to block NNK-induced FAK phosphorylation and ERK activation, resulting in an overall lower proliferation and migration (50 µM) [180], and to inhibit the ERK-FAK pathway leading to a repression of the cells’ migratory ability [156].

Anthocyanins are found to have an inhibitory effect on phospho-Akt (>15 µM) [181] and MAPK signaling pathways (>50 µM) [182], to lower FAK expression and interaction with HER-2 (>100 µM) [183] and to inhibit the PI3K/Akt/NF-κB pathway (>40 µM) [184]. In particular, cyanidin-3-*O*-sambubioside and delphinidin lowered the expression of phospho-Akt or blocked the MAPK signaling pathway, inducing a decrease in MMP-9 and leading to the inhibition of the metastatic processes in breast cancer cells [181,182]. In the same tumor cell type, black rice anthocyanins were able to decrease the interaction between HER-2 and FAK [183]. Further, anthocyanins from blueberry extract modulated the PI3K/Akt/NF-κB pathway, resulting in inhibition of growth and metastatic potential of different cancer cells [184]. Naringin (>600 µM) suppressed EGFR and ERK phosphorylation levels in HeLa and in the A549 lung cancer cell line [185]. Furthermore, naringenin and naringin (50 µM) suppressed TPA-induced AP-1 activity by inhibiting the phosphorylation of ERK and c-Jun N-terminal kinase (JNK) in HepG2, Huh-7, HA22T and BNL CL2 cell lines [186]. They also suppressed TPA-induced DNA-binding and activation of ERK/PI3K/Akt upstream of NF-κB and AP-1. Lastly, naringenin and hesperetin (10 µM), administered alone or in combination, inhibited the phosphorylation of FAK and p38 signaling in vitro and in vivo in human pancreatic cancer [187].

Similarly, the isoflavone genistein inhibited FAK phosphorylation in pancreatic cancer (60 µM) and hepatocellular carcinoma cells (10 µM) [188,189]. In addition to inhibiting FAK phosphorylation, genistein (>200 nM) also inhibited HSP27 phosphorylation, MMP-2 induction and cell invasion, by blocking phosphorylation (i.e., activation) of p38 MAPK in human PCa cells [190]. Moreover, genistein (>25 µM) inhibited p38 and MAPK activation in other cancer cell lines [191].

Numerous studies showed that also the non-flavonoid compounds coumarins actively interact with intracellular protein of FAPs. Osthole (15 µM) decreased the phosphorylation of FAK and cell motility in human glioma cells [192]. 4-hydroxycoumarin (4-HC) treatment (500 µM) caused a decrease in β-paxillin protein and mRNA levels, a decrease in FAK phosphorylation, the inhibition of the capacity to originate pulmonary metastases and the downregulation of Adhesion Regulating Molecule-1 (ARM-1) in melanoma cells [193]. Farnesiferol C (FC) decreased the expression of CD34, Ki-67 and of phosphorylation of most of the kinases downstream of VEGFR2 (FAK, Src, ERK1/2, p38 MAPK and c-Jun-NH2-kinase) in HUVEC cells [194]. In addition, gossypol (20 µM), a small BH3-mimetic polyphenol extracted from cotton seeds, was able to inhibit the activation of ERK1/2 and Akt and to stimulate activation of p38 and JNK1/2, required for apoptosis induction in head and neck carcinoma cells [195].

Many studies investigated the effects of curcumin on FAK and the associated signaling [196]. Curcumin affected v-Src kinase and FAK enzymatic activity and Akt phosphorylation [197,198]. The study by Lin et al. showed that curcumin (15 µM) is able to decrease protein kinase C (PKC), FAK, NF-κB p65 and Rho-A protein levels, leading to the inhibition of ERK1/2, MKK7, COX-2 and ROCK1 in mouse–rat hybrid retina ganglion cells (N18) [199]. Further, Chen et al. evaluated the effects of curcumin in human colon cancer cell lines from the NCI-60 panel (including HCT-116, HT-29, HCT-15, HCC-2998, Colo205, Km-12 and SW-620 cells), both in vitro and in SCID mice, and found that this polyphenol inhibits FAK phosphorylation and decreases CD24 expression [161]. Further, curcumin (>10 µM) decreased the protein levels of FAK, phospho-FAK, Rac1 and Cdc42 in mouse neuroblastoma N18 and rat glioma C6 cell lines [200]. Treatment with curcumin (11.5 µM) altered the phosphorylation of several kinases such as TNK2, FRK, AXL and MAPK12 and phosphatases such as PTPN6, PTPRK and INPPL1 in the Cal-27 cell line [201]. A similar finding was observed in the study by Choe et al., in which curcumin caused the inhibition of the RCP-induced EGFR/FAK phosphorylation in ovarian cancer cells [202]. Curcumin in combination with exposure to light also suppressed phospho-FAK expression in bladder carcinoma cells [162]. In addition, a combined treatment with curcumin (>50 µM) and wikstroflavone B (WFB) (>100 µM) inhibited viability, migration, colony formation and invasion in four human nasopharyngeal carcinoma (NPC) cell lines, by modulating several proteins, such as survivin, cyclin D1, p53 and p21, STAT3 and FAK [203].

As for the class of phenolic acids, there are several works demonstrating their involvement in the regulation of intracellular proteins. For example, CAPE (20 µM) was demonstrated to be able to inhibit FAK phosphorylation and the downstream p38 and c-Jun N-terminal kinase (JNK) signaling pathways in a human tongue squamous carcinoma cell line (SCC-9) [204]. A reduction in FAK phosphorylation and paxillin inhibition was also observed in the SNU-1041 cell line upon CAPE treatment (4 µM) [164]. Similarly, caffeic acid 3,4-dihydroxyphenethyl ester (CADPE) caused the inhibition of AP-1 and c-fos nuclear factor activity as well as the inhibition of PMA-induced FAK, ERK and MEK phosphorylation in human gastric and breast carcinoma cell lines (25 µM) [205]. Ferulic acid (FA) inhibited SMAD and FAK activity in an immortalized rat cell line (HSC-T6) (30 µM) [206]. Another phenolic acid, GA (>50 µM), was reported to have dose-dependent effects in the regulation of intracellular proteins in prostate cancer: these included the inhibition of growth factor receptor-bound protein 2 (GRB2), PKC, NF-κB p65, JNK, ERK1/2, p38 and phospho-Akt. Moreover, it caused the increase in PI3K and Akt and in *TIMP-1* gene levels but also the inhibition of FAK and Rho-A mRNA levels [207]. A similar mechanism of action is exerted by methyl gallate (MG), which was able to reduce viability, migration, Akt and ERK1/ERK2 phosphorylation and to inhibit paxillin phosphorylation and focal adhesion turnover in the rat C6 glioma cell line (5 µg/mL) [208]. Bornyl cis-4-hydroxycinnamate also induced the inhibition of the FAK/PI3K/Akt/mTOR, MAPK and GRB2 signaling pathways in A2058 and A375 cell lines, according to the work by Yang et al. [163]. In addition, ellagic acid (EA) (30 µM) induced the accumulation of p53, the inhibition of Akt and also the increase in the PTEN phosphatase activity in the mouse melanoma cell line B16F10 [209]. A decrease in FAK and Akt phosphorylation was also observed in the study on the effects of *Crataegus* berries, leaves and flowers compounds, carried out in U87MG human glioblastoma cells (250 µg/mL) [210]. The chloropyramine–cinnamic acid hybrids (10 µM) also inhibited FAK autophosphorylation and reduced focal adhesion formation and stress fibers in the MDA-MB-231 cell line [211]. Mostafa et al. tested the effect of plasma anthocyanins and their metabolites in PANC-1 and AsPC-1 cell lines and found that these molecules induce the reduction in NF-κB, FAK and p65 activity [172].

The class of lignans is involved in the regulation of intracellular proteins through various mechanisms. In particular, magnolol (20 μM) inhibited NF-κB signaling, NF-κB activity, phospho-p65 and phospho-IκBα in breast cancer cell lines in vitro and in vivo [212]. Further, nordihydroguaiaretic acid (NDGA) (>10 μM) was able to inhibit cell migration by suppressing NRP1 expression and function, leading to attenuated cell motility, cell adhesion to ECM and FAK signaling in prostate cancer cells in vitro and in vivo [213].

Several works showed that resveratrol suppresses tumor cell growth by acting as a FAK inhibitor. Resveratrol-mediated antitumor effects are enhanced by inhibition of FAK and cytoskeleton proteins, as demonstrated in human colorectal cancer SW480 and HCT116 cell lines (5 μM) [214] and in the colon cancer HT29 cell line (100 μM) [215]. Resveratrol (25 μM) also inhibited cell migration, increasing filopodia formation and decreasing the number of focal adhesions and FAK activity in the human breast cancer cell line MDA-MB-231 [216].

In conclusion, the different classes of polyphenols act in the tumor area on multiple intracellular proteins involved in focal adhesion plate turnover by inhibiting phosphorylation upstream of the FAK protein and preventing its activation, as well as by inhibiting the activation of downstream proteins, such as ERK and Akt, thus causing a blockade of signal transduction pathways involved in cell survival and migration (Table 1).

### 5.3. Effects of Polyphenols on Cytoskeleton

Different studies have reported that polyphenols are able to affect cytoskeleton organization in normal and cancer cells. An earlier study by Medrano and Andreu reported that gossypol inhibits the in vitro assembly of microtubules without their distortion. Thus, the authors proposed that gossypol may act as a new tubulin ligand [217,218]. Later, Hu et al. reported that apigenin is able to reduce the expression of FAK by affecting its protein stability in ovarian cancer cells and that the decrease in FAK was paralleled by the disruption of actin polymerization and by the decrease in stress fibers. Indeed, the actin fibers were located compactly in the cell periphery upon apigenin treatment rather than in the inner part of the cell, as observed in untreated control cells. Accordingly, apigenin suppressed the in vitro migration and invasion of human ovarian cancer cells and their ability to induce metastasis in nude mice [178]. Similarly, Franzen et al. showed that apigenin reduces FAK/Src signaling in prostate cancer cells and that such effect was accompanied by actin cytoskeleton remodeling and by the appearance of exaggerated filopodia, which stimulated a strong attachment of cancer cells to an adhesive surface. Consistent with these findings, both motility and invasiveness of cancer cells were decreased by the treatment [179]. The effect of curcumin on cytoskeletal organization was studied by Kim et al., who reported that the treatment of chick limb bud mesenchymal cells with this compound significantly increased actin stress fibers. This effect was paralleled by the decreased expression of β1 integrin and by the reduction in FAK phosphorylation. The modulation of the actin cytoskeleton was supposed to be dependent on Akt signaling inactivation [198]. Different studies have demonstrated that curcumin is able to bind microtubules and to suppress their dynamic changes in cancer cells. In fact, curcumin depolymerized mitotic spindle microtubules in breast cancer cells [219,220] and interphase and mitotic spindle microtubules in cervical and breast cancer cells [220]. In addition, Lee et al. showed that cervical cancer cells’ death induced by vinblastine was inhibited by the pre-treatment with curcumin, which diminished microtubules depolymerization [221]. Inhibition of FAK by EGCG and the ensuing modification of cytoskeleton organization was reported in benign prostatic hyperplasia cells. The authors demonstrated that EGCG disrupted the organization of F-actin and decreased paxillin distribution. The effect of EGCG on the disorganization of actin cytoskeleton was mirrored by the decreased cells’ migration [222]. EGCG also induced cytoskeleton reorganization by rescuing the abnormal actin organization promoted by the protease-activated receptor 2 agonist peptide (PAR2-AP) or by factor VIIa in human colon cancer cells and inhibited cell migration [223]. The ability of EGCG to inhibit FAK phosphorylation and actin cytoskeleton organization during cells’ adhesion was also reported in fibroblasts which, in the presence of EGCG, reduced their motility as well [224]. To further support the effect of polyphenols in modulating the organization of the cytoskeleton, Chen et al. reported that resveratrol decreased the expression of α-smooth muscle actin (α-SMA) and the level of β-catenin, which indirectly anchors the cadherins to the cytoskeleton in leiomyoma cells [225]. The effect of resveratrol on the cytoskeleton was further investigated by Azios et al., who showed that resveratrol sustains the extension of actin structures like filopodia in breast cancer cells in a time- and concentration-dependent modality. At low concentration (5 µM), resveratrol increased rapid, sustained leading-edge lamellipodia by enhancing Rac activity and thus promoted cell migration and invasion. On the other hand, resveratrol at high concentration (50 µM) induced the rapid extension of unpolarized filopodia by the inhibition of Rac and Cdc42 activity and thus inhibited cell migration [226]. A high concentration of resveratrol disturbed cytoskeleton remodeling and ROCK1 in human tubular epithelial cell as well [227]. The resveratrol derivative (Z)-3,5,4′-trimethoxystilbene was shown to prevent tubulin polymerization in colon cancer cells [228]. On the other hand, resveratrol and its analogs were extensively reported to be able to reverse EMT, which involves cytoskeleton reorganization in different types of tumors [171,229].

### 5.4. Effects of Polyphenols on MMPs

MMPs are involved in the degradation of ECM components such as proteins (collagen, vitronectin and fibronectin) and glycosaminoglycans. In a tumor context, MMPs are often overexpressed or their enzymatic activity is increased, thus allowing tumor cells to invade surrounding tissues more efficiently. Several studies reported the effects of flavonoids on the activity of MMPs.

Quercetin, a polyphenol contained in numerous foods such as capers, red onions, red apples, grapes and cabbage, was demonstrated to inhibit metastasis formation and EMT in pancreatic cancer cell lines, epidermoid carcinoma cells and squamous cell carcinoma cells of the tongue, by decreasing the secretion of MMPs and gelatinases [173,175,176]. Hung et al. observed the decrease in the invasive capacity of renal adenocarcinoma cells upon kaempferol treatment in vitro and in vivo. This effect was achieved through a strong inhibition of MMP-2 activity, due to an inhibition of the PI3K/Akt signaling pathway [174]. Similar effects were demonstrated for EGCG (10 µM). Chen et al. observed a dose-dependent decrease in the invasive capacity of squamous carcinoma of the tongue cells through an inhibition of the synthesis of MMPs and plasminogen activator (uPA), with a significant decrease in interactions between cells and ECM [230]. In another study, Sen et al., by investigating the antitumor effects of EGCG in different tumor cell lines [MCF7 (breast cancer), A375 (melanoma) and HT-1080 (fibrosarcoma)], observed a dose-dependent decrease in MMP-2 mRNA levels and activity and also a greater difficulty of the cells in forming bonds with ECM components such as vibronectin and vitronectin [154]. Similarly, the anthocyanin delphinidin was shown to lower MMPs by acting at the transcriptional level and inhibiting the activity of NF-κB in MCF-7 breast cancer cells [182].

Mulberry anthocyanins, cyanidin 3-rutinoside and cyanidin 3-glucoside, are capable of inhibiting the expression of MMPs, in particular MMP-2, and plasminogen activator-urokinase (PAI) in metastatic lung cancer and melanoma cells (A549, B16-F1) [231,232]. A similar effect was observed by Lee et al. upon treatment of breast cancer cells (MDA-MB 231) with cyanidin-3-*O*-sambubioside, associated with the reduction in the gelatinolytic activity of MMPs, in particular MMP-9 [181]. The decrease in MMP-9 activity along with MMP-1 or MMP-2 was observed in different tumor cell lines (HCC38, HCC1937, MDA-MB 231, A549) after treatment with blueberry extract [184] or with the flavanone naringenin (100 µM) [233]. Yen et al. also demonstrated how naringin is able to inhibit the synthesis of MMP-9 at the transcriptional level by reducing the activity of AP-1 and NF-κB in hepatocarcinoma cells (Hep-G2, Huh7, HA22T, and BNLCL2) [186]. Xu et al. studied the combined effect of naringenin (1–1000 µM) with tamoxifen on MCF-7 human breast adenocarcinoma cells, observing an overall dose-dependent downregulation of MMP-2 and MMP-9 expression [234]. A decrease in the induction of MMP-2 and cell migration following a block in the phosphorylation of MAP kinase p38 was observed upon in vitro and in vivo treatment of prostate cancer cells with the isoflavone genistein [190,191].

Numerous studies showed that the non-flavonoid compounds have an important impact on the action of several enzymes involved in the degradation of the ECM such as MMPs, thus influencing cell invasiveness and motility. Velasco-Velázquez et al. demonstrated how 4-hydroxycoumarin (4-HC) inhibited the formation of lung metastases by murine melanoma cells (B16-F10) [193]. Twarock and his team observed that 4-methylumbelliferone (4-MU) (300 µM) was able to inhibit filopodia, focal adhesion formation and cell migration in human esophageal carcinoma cell lines [235]. Lee et al. reported that FC used in different concentrations on HUVECs and in vivo on Sprague–Dawley rats is capable of inhibiting VEGF-induced cell migration as well as the expression of MMP-2 [194]. Studying the effect of osthole on human glioma cells, Tsai and his research group found that osthole is able to inhibit the expression of MMP-13 in a dose-dependent manner, thus lowering the cells’ ability to migrate [192]. Umbelliprenin also appeared able to inhibit the catalytic activity of certain MMPs such as MMP-2 and MMP-9 in vivo and thus the formation of metastases, as demonstrated by Naderi Alizadeh et al. [160]. In addition, studying the effect of polyphenols on ovarian (HeLa and A2780) and cervix (SiHa and HeLa) tumor cell lines, Jamialahmadi et al. and Ying et al. found that auraptene (>12.5 μM) and angelol-A (120 µM) were able to inhibit the expression of MMP-2 and MMP-9, lowering the invasive capacity of the two tumor cell types [236,237].

One of the polyphenols that has certainly been most studied in the medical field is curcumin. Specifically, in evaluating the action of curcumin on the invasive capacity of tumor cell lines, Lin et al. and Thiyagarajan et al. demonstrated how even low concentrations of curcumin are already able to inhibit the enzymatic action of MMP-2 and MMP-9 in a mouse–rat hybrid retina ganglion cell line (N18) [199,200]. Shao and his collaborators found that curcumin and the biflavonoid wikstroflavone B, alone and in combination, negatively modulated the synthesis of several proteins, such as MMP-2 and MMP-9, thus blocking cell migration in four human NPC cell lines (CNE1, CNE2, HONE1 and C666-1) [203].

Similarly, the caffeic acid derivative CAPE (50 µM) was able to dose-dependently decrease MMP-2 and MMP-9 synthesis and enzymatic activity in human fibrosarcoma cells (HT1080) [238]. The same effect was observed by Lee and his research team using CAPE on human hepatocarcinoma SK-HEP1 cells (12.5 µM) [239]. Peng et al. demonstrated that CAPE also lowers the catalytic activity of MMP-2 in human tongue squamous carcinoma SSC-9 cells [204]. Another caffeic acid ester, CADPE, was studied by Han et al., who demonstrated that it is able to reduce the invasive capacity of human gastric carcinoma and breast carcinoma cells by inhibiting the PMA-dependent MMP-9 activation pathway [205]. A similar inhibition of MMP-2 and MMP-9 activities was found upon treatment with a caffeic acid derivative, PT93 (>30 µM), on immortalized rat HSC-T6 cells [240]. Liu et al. instead focused their studies on another compound belonging to the class of phenolic acids (GA) and demonstrated that GA treatment inhibited MMPs’ activity in prostate carcinoma cells (PC-3) by increasing the synthesis of the inhibitor TIMP-1 [207]. A GA derivative, methyl gallate (MG), was employed to treat C6 rat glioma cells, resulting in an inhibition of adhesion plate turnover and thus a decrease in cell migration [208]. Another phenolic acid, bornylcis-4-hydroxycinnamate, was demonstrated to be able to decrease the expression of MMP-2 and MMP-9 by inhibiting FAK/PI3K/Akt/mTOR, MAPK and GRB2 signaling pathways in melanoma cells [163]. Yang et al., employing a hybrid of cinnamic acid and chloropyramine on breast carcinoma cells (MDA-MB-231), showed that this compound is able to inhibit the formation of focal adhesions [211].

Within the class of lignans, the most studied polyphenol is magnolol. Several studies reported the decrease in invasiveness and MMP-2/MMP-9-mediated migration in several breast cancer cell lines [212], in a cholangiocarcinoma cell line (30 μM) [241] and in a prostate cancer cell line (>5 μM) [242]. Another lignan, honokiol (>7.5 μM), dose-dependently decreased the number of lung cancer H1299 cells capable of invading the surrounding tissue through an inhibition of MMP-2/-9 [243].

Similar effects were reported for the stilbenes resveratrol and pterostilbene. The combined treatment with resveratrol and pterostilbene (>25 μM) resulted in a greater inhibition of the expression of MMP-2 and MMP-9, accompanied by a strong reduction in cell growth in three different cervical cancer cell lines (HeLa, SiHa and CaSki) [244]. Further, it has been reported that resveratrol is able to increase filopodia formation, inhibit cell migration and decrease the percentage of focal adhesions in a dose-dependent manner in breast cancer cells [216]. Pterostilbene (10 μM) was also studied by Pan and his team, who demonstrated inhibition of MMP-9 upon treatment of breast cancer cells [245]. Similar effects were induced by the xanthones α-mangostin and mangiferin. α-mangostin (>7.5 μM) was found to inhibit MMP-2 and MMP-9 in pancreatic cancer cells [165]. Mangiferin (200 μM) suppressed the TNF-α-induced expression of MMP-9 by inhibiting the transcription factor NF-κB in prostate cancer cells [246]. Moreover, mangiferin inhibited the growth and invasiveness of ER-positive and ER-negative breast cancer cells in vitro and in vivo by lowering the expression of MMP-7 and MMP-9 and by reducing the EMT [168]. A similar effect was observed in astroglioma, melanoma and ovarian cancer cells upon treatment with mangiferin (100 μM), with a global inhibition of MMP-1, -2, -7, -9, -14 and of EMT [247,248]. An overall reduction in the expression of MMP-2 and MMP-9 was also observed by Luo and his team upon treatment of glioma cells with gartaninin [249]. An inhibition of cell migration and a downregulation of the FAK/MMP-dependent signaling pathway were observed after treatment with cratoxylumxanthone C in A549 lung cancer cells (7.5–30 μM) [250].

In conclusion, polyphenols are able to decrease the enzymatic activity of MMPs and in some cases can also act at the gene level, leading to a reduction in MMPs’ expression (Table 1). 

**Table 1:** Effects of polyphenols on the modulation of FAPs components and tumor cell’s invasive behavior.

Polyphenol	In Vitro Model	In Vivo Model	Effect on FAP	Ref.
Flavonoids				
*Flavonols*				
Quercetin	Human pancreatic cancer cell lines (PATU-8988) (20–40–80–160 µM)		↓ Invasion and metastasis ↓ STAT3 signaling pathway ↓ IL-6-induced EMT and MMP secretion	[175]
	Human medulloblastoma cell line (DAOY); human hepatoma cell line (HepG2); human melanoma cell lines (A375, A2058) (20–40–60–80 µM)		↓ Migration and invasion ↓ Activation of c-Met and downstream molecules ↓ FAS	[177]
	Human pancreatic cancer cell line (MiaPaCa-2) and skin tumor cell line (A431) (10–20–50–100 µM)		↓ EGFR tyrosine kinase activity and its signal pathway ↓ MMP-9/-2 enzymatic activity ↓ FAK protein phosphorylation	[173]
	Human oral squamous cell carcinoma cell line (SAS) (25–50-100–200–400 µM)		↓ Migration and invasion ↓ MMP-9/-2 enzymatic activity ↓ FAK, p-ERK1/2	[176]
Kaempferol	Human renal cell adenocarcinoma cell line (786-O) and human proximal tubule epithelial cell line (HK-2) (25–50–75–100 µM)	SCID mice i.v. inoculated with 1 × 10^6^ 786-O cells and treated with 2–10 mg/kg of kaempferol by oral gavage	↓ Cell invasion and migration ↓ MMP-2 expression ↓ FAK phosphorylation ↓ PI3K/Akt pathway ↓ Tumor mass in mice	[174]
*Flavan-3-ols*				
Epigallocatechin-3-gallate (EGCG)	Human benign prostate hyperplasia cell line (BPH-1) (from 1 to 100 µM)		↓ Cell migration ↓ Actin cytoskeleton organization and paxillin distribution ↓ Focal adhesion proteins	[222]
	Human pancreatic cancer cell lines (AsPC-1, BxPC-3) (25–40–75–80–100 µM)		↓ Cell adhesion ↓ FAK and IGF-1R activation	[153]
	Human tongue squamous cell carcinoma cell line (SCC-9) (5–10–15–20 µM)	BALB/c nu/nu mice s.c. inoculated with 1 × 10^7^ SCC-9 cells and treated with 10–20 mg/kg of EGCG by oral gavage	↓ Cell invasion and motility ↓ Cell–matrix interaction ↓ MMP-2 expression and activity ↓ FAK phosphorylation ↓ NF-κB and Snail-1 levels ↓ u-PA expression ↓ PMA-induced MMP-9 expression ↓ Tumor mass in mice	[230]
	Human breast cancer cell line (MCF-7), human melanoma cell line (A375) and human fibrosarcoma cell line (HT-1080) (5–10–20–40 µM)		↓ MMP-2 enzymatic activity and mRNA levels ↓ MT1-MMP expression ↓ Binding with the extracellular proteins (vitronectin and fibronectin) ↓ Integrin receptor expression ↓ FAK expression ↓ ERK phosphorylation ↓ VEGF expression	[154]
*Flavones*				
Apigenin	Human pancreatic cancer cell lines (PC3-M, C4-2B, DU145) (10–25–50 µM)		↓ Cell motility ↑ Filopodia and matrix attachment ↓ Actin structures formation during migration ↓ FAK/Src activation	[179]
	Human pancreatic cancer cell lines (BxPC-3, MIAPaCa-2) (50 µM)		↓ NNK-induced pancreatic cellular proliferation ↓ NNK-induced FAK phosphorylation ↓ NNK-induced ERK activation	[180]
	Human melanoma cell lines (A2058, A375) (10–20–50 µM)		↓ Integrin subunits expression ↓ ERK phosphorylation ↓ Cell migration	[156]
	Human ovarian cancer cell line (A2780) (20–40 µM)		↓ Cell migration and invasion ↓ Actin organization and focal adhesion formation ↓ FAK phosphorylation and expression	[178]
Luteolin	Human pancreatic cancer cell line (MiaPaCa-2) and skin tumor cell line (A431) (10–20–50–100 µM)		↓ EGFR tyrosine kinase activity ↓ MMP-9/-2 secretion ↓ FAK phosphorylation and expression levels	[173]
*Anthocyanins*				
Mulberry Anthocyanins, Cyanidin 3-rutinoside and Cyanidin 3-glucoside	Highly metastatic human lung carcinoma cell line (A549) (25–50–100 mM)		↓ MMP-2 ↓ u-PA ↑ TIMP-2 ↑ plasminogen activator inhibitor (PAI)	[231]
Mulberry Anthocyanins (MACs)		C57BL/6 mice inoculated via a right groinal injection with melanoma B16-F1 cells and treated with food administration of PBS plus 1–2 or 3% of MACs	↓ MMP-2/-9 expression	[232]
Cyanidin-3-*O*-sambubioside	Human breast cancer cell line (MDA-MB-231) (1–10–30 µM)		↓ p-Akt and MMP-9 activity and expression level	[181]
Delphinidin	Human breast cancer cell line (MCF-7) (15–30–60–90 µM)		↓ *MMP-9* gene transcriptional activity by blocking the activation of NF-κB through MAPK signaling pathways	[182]
Black Rice Anthocyanins	Human breast cancer cell lines (MCF-7, MDA-MB-453) (100–200–300–400–500 µM)		↓ Interaction between HER-2 and FAK, FAK and cSrc, cSrc and p130 Cas, and FAK and p130 Cas ↓ Phosphorylation of FAK, cSrc and p130 Cas	[183]
Blueberry Extract	Human breast cancer cell lines (HCC38, HCC1937, MDA-MB-231) (10–20–40–80 µM)		Inhibition of MMP-1 and plasminogen activator inhibitor-1 secretion ↑ u-PA secretion ↓ MMP-9 and PI3K/Akt/NF-κB pathway	[184]
*Flavanones*				
Naringenin	Human lung cancer cell line (A549) (25–50–100–200–300 µM)		↓ MMP-2/-9 enzymatic activity	[233]
	Human prostate cancer cell line (PC-3) (25–50–100–200–300 µM)		↓ Cell migration and invasion ↓ u-PA, SNAI1, SNAI2 and TWIST1 activity ↑ E-cadherin	[159]
Naringin	Human cervical cancer cell line (HeLa) and human lung cancer cell line (A549) (400–3200 µM)		↓ EGFR and ERK phosphorylation levels	[185]
		Apc^Min/+^ mice treated with 150 mg/kg of naringin by gavage	Modulation of the activity of GSK-3β and inhibition of β-catenin expression in intestinal adenomatous cells	[158]
Naringenin and Naringin	Human hepatocellular carcinoma cell lines (HepG2, Huh-7, HA22T, BNLCL2) (25–50–100 µM)		↓ MMP-9 transcription by inhibiting AP-1 and NF-κB activity ↓ ERK and JNK signaling pathways	[186]
Naringenin and Hesperetin	Human pancreatic cancer cell lines (Panc-1, MiaPaCa2) (1–5–10–20 µM)	BALB/c nude mice s.c. inoculated with 10^7^ Panc-1 cells and treated with 10–30 mg/kg of naringenin and hesperetin alone and in combination	↓ FAK phosphorylation ↓ p38 signaling pathway	[187]
Naringenin and Tamoxifen	Human breast cancer cell line (MCF-7) (Tamoxifen 0.001–50 µM, Naringenin 1–1000 µM)		↓ MMP-2/-9 expression levels	[234]
*Isoflavones*				
Genistein	Human pancreatic cancer cell lines (AsPC-1, BxPC-3, Capan-2) (60 µM)		↓ FAK phosphorylation	[188]
	Human hepatocellular carcinoma cell line (MHCC97-H) (5–10–20 µM)	Male athymic BALB/c nu/nu mice s.c. inoculated with MHCC97-H cells and treated i.p. with 50 mg/kg of genistein	↓ FAK expression and phosphorylation	[189]
	Human prostate cancer cell line (PC3-M) (1–10.000 nM)	Male athymic BALB/C mice (orthotopic implantation of PC3-M cells) treated with 100–200 mg/kg of genistein administered with food	↓ FAK phosphorylation ↓ HSP27 phosphorylation ↓ MMP-2 induction and cell invasion by blocking p38 phosphorylation	[190]
	Human prostate cancer cell lines (PC-3, PC3-M, DU-145) (1–50 µM)		↓ MMPs activity ↓ Cell invasion ↓ p38 activation	[191]
Non-flavonoids				
*Coumarins*				
4-Hydroxycoumarin (4-HC)	Murine melanoma cell line (B16-F10) (500 µM)		↓ β-paxillin mRNA expression levels ↓ FAK phosphorylation ↓ Lung metastasis ↓ ARM-1	[193]
4-Methylumbelliferone (4-MU)	Human esophageal squamous carcinoma cell line (OSC1) (300 µM)		↓ Filopodia and focal adhesion formation	[235]
Osthole	Human glioma cells (U251, HS683) (1–10–30 µM)		↓ MMP-13 expression levels ↓ FAK phosphorylation ↓ Cell motility	[192]
Umbelliprenin		BALB/c mice s.c. inoculated with 1 × 10^5^ colorectal cancer cells CT26 and treated i.p. daily with 12.5 mg/kg of umbelliprenin	↑ IFN-γ levels ↓ IL-4 levels ↑ E-cadherin levels ↓ Ki-67 levels ↓ MMP-9/-2 expression levels ↓ VEGF levels ↓ Lung and liver metastasis	[160]
Auraptene	Human ovarian cancer cell line (A2780) and human cervical cancer cell line (HeLa) (0.78125–1.5625–3.125–6.25–12.5–25–50–100 μM)		↓ Invasion and migration ↓ MMP-2/-9 enzymatic activity	[236]
Angelol-A	Human cervical cancer cell line (SiHa and HeLa) and human proximal tubular (PTC) cell line (HK2) (40–80–120–160–200 µM)		↓ MMP-2 and VEGF-A expression by ↑ expression of miR-29a-3p (that targets the VEGFA-3′ UTR)	[237]
*Curcuminoids*				
Curcumin	Mouse–Rat hybrid retina ganglion cell line (N18) (7.5–15 µM)		↓ PKC, FAK, NF-κB p65 and Rho A protein levels ↓ ERK1/2, MKK7, COX-2 and ROCK1 ↓ MMP-2 and MMP-9	[199]
	Human colon cancer cell lines from NCI-60 panel (HCT-116, HT-29, HCT-15, HCC-2998, Colo205, Km-12, SW-620) (10–20–30–40–50 µM)	SCID mice inoculated in the spleen with 1 × 10^6^ HCT-116 cells and treated with 1 g/kg of curcumin administered daily by gastric intubation	↓ Sp-1 transcriptional activity and Sp-1 regulated genes (*ADAM10*, *CALM1*, *EPHB2*, *HDAC4* and *SEPP1*) ↓ Inhibition of FAK phosphorylation ↓ CD24 expression ↑ E-cadherin expression	[161]
	Mouse neuroblastoma cell line (N18) and Rat glioma cell line (C6) (2.5–5–10–25–50 µM)		↓ Formation of filopodia on the intracellular surface ↓ MMP-2 and MMP-9 enzymatic activity ↓ Protein levels of FAK, pFAK, Rac1 and Cdc42	[200]
	Human tongue squamous cell carcinoma cell line (Cal-27) (11.5 µM)		↓ Phosphorylation of several kinases (TNK2, FRK, AXL, MAPK12) and phosphatases (PTPN6, PTPRK and INPPL1)	[201]
Curcumin plus visible light	Human bladder carcinoma cell lines (RT112, UMUC-3) (0.27–0.54–1.08 µM)		↓ FAK phosphorylation ↓ Integrin expression	[162]
Curcumin and Wikstroflavone B (WFB)	Four human NPC cell lines (CNE1, CNE2, HONE1, C666-1) (20–40–60–80–100 µM CUR, 100–200–300–400 µM WFB)		↓ Cell migration, invasion, colony formation and viability by modulating several proteins (Survivin, cyclin D1, p53 and p21, MMP-2, MMP-9, STAT3 and FAK)	[203]
*Phenolic acids*				
Caffeic Acid 3,4-Dihydroxyphenethyl Ester (CADPE)	Human gastric carcinoma cell lines (MGC-803, HGC-27, AGS) and human breast carcinoma cell line (MDA-MB-231) (1–5–10–25–50 µmol/L)		↓ PMA-induced increase in MMP-9 activity ↓ c-fos and AP-1 nuclear factor activity ↓ PMA-induced FAK, ERK and MEK phosphorylation	[205]
Caffeic Acid Phenethyl Ester (CAPE)	Human fibrosarcoma cell line (HT1080) (20–50–80–100 µM)		↓ MMP-2/-9 enzymatic activity ↓ MMPs mRNA levels	[238]
	Human hepatocellular carcinoma cell line (SK-Hep1) (6.25–12.5–25 µM)		↓ MMP-2/-9 activity ↓ NF-κB DNA-binding activity	[239]
	Human tongue squamous carcinoma cell line (SCC-9) (5–10-20–40 µM)		↓ FAK phosphorylation ↓ p38 and c-jun signaling pathways ↓ MMP-2 activity	[204]
	Human hypopharyngeal squamous cell carcinoma (SNU-1041) (4 µM)		↓ EMT progression ↑ E-cadherin expression ↓ FAK phosphorylation ↓ Paxillin expression	[164]
Gallic Acid (GA)	Human prostate cancer cell line (PC-3) (25–50–100–150 µM)		↓ MMP-2/-9 activity ↓ GRB2, PKC, NF-κB p65, JNK, ERK1/2, p38, p-Akt ↑ PI3K and Akt inhibition of FAK and Rho-A mRNA levels ↑ *TIMP-1* gene levels	[207]
Methyl Gallate (MG)	Rat glioma cell line (C6) (0.1–0.5–1–5–10–20 µg/mL)		↓ Cell viability, invasion and migration ↓ Akt phosphorylation levels ↓ ERK1/2 phosphorylation ↓ Paxillin phosphorylation and focal adhesion turnover	[208]
Ferulic Acid (FA)	Immortalized rat cell line (HSC-T6) (1–3–10–30–100–300 µM)		↓ α-1 collagen and fibronectin expression ↓ SMAD activity ↓ FAK activity	[206]
PT93 (a novel caffeic acid amide derivate)	Human malignant glioblastoma cell lines (T98G, U87, U251) and normal mouse neuron cells (HT22) (1–3–10–30–100–200–300 µM)		↓ MMP-2/-9 activity	[240]
Bornyl cis-4-Hydroxycinnamate	Human melanoma cell lines (A2058, A375) (1–3–6–12–18–24–32–36 µM)		↓ MMP-2 and MMP-9 expression through inhibition of FAK/PI3K/Akt/mTOR, MAPK and GRB2 signaling pathways ↓ EMT progression	[163]
Ellagic Acid (EA)	Mouse melanoma cell line (B16-F10) (15–30 µM)		↑ p53 accumulation ↑ PTEN phosphatase activity ↓ Akt activity	[209]
Ethanolis extract of *Ocinum sanctum* leaves (EEOS)	Head and neck squamous cell carcinoma cell lines (HN4, HN12, HN30, HN31) (0.05–0.1–0.2–0.4–0.8 mg/mL)		↓ MMP-2/-9 activity	[251]
Extracts of *Crataegus* berries, leaves, and flowers compounds	Human glioblastoma cell line (U87MG) (100–250-500 µg/mL)		↓ FAK and Akt phosphorylation	[210]
Chloropyramine-cinnamic acid hybrids	Human breast cancer cell line (MDA-MB-231) (5–10–20 µM)		↓ FAK Y925 phosphorylation ↓ Focal adhesion formation	[211]
Plasma anthocyanins and their metabolites	Human pancreatic cancer cell lines (PANC-1, AsPC-1)		↓ β1- and β4-integrins and intercellular adhesion molecule-1 ↓ NF-κB p65 and FAK activity	[172]
*Lignans*				
Magnolol	Human breast cancer cell line (MDA-MB-231) (10-20-30 μM)	Female (nu/nu) mice s.c. inoculated with MDA-MB-231 (6 × 10^6^) or MCF-7 (4 × 10^6^) cells and treated i.p. with 40 mg/kg of magnolol four times a week	↓ MMP-2/-9 activity ↓ NF-κB activity ↓ p65 and p-IKB activity	[212]
	Human cholangiocarcinoma cell line (MZ-ChA-1) (10–20-30–40 μM)		↓ MMP-2/-7/-9 levels	[241]
	Human colorectal adenocarcinoma cell lines (HCT116, SW480) (2.5–5–10 μM)		↑ Epithelial markers (E-cadherin, ZO-1, claudin) ↓ Mesenchymal markers (N-cadherin, TWIST-1, Slug and Snail)	[166]
	Human glioblastoma cell lines (U87MG, LN229) (20–40–60 μM)	BALB/cAnN.C-Foxn1nu/CrlNarl nude mice (10^5^ LN229-Luc2 cells implanted in the right cerebral hemisphere) treated i.p. with 20 mg/kg/day of magnolol for 10 days	↓ Focal adhesion formation ↓ N-cadherin expression	[167]
	Human prostate cancer cell line (PC-3) (2.5–5–10–20 μM)		↓ Cell migration by attenuating MMP-2/-9 expression	[242]
Honokiol	Human non-small-cell lung carcinoma cell line (H1299) (2.5–5–7.5–10 μM)		↓ Cell migration ↓ Cell invasion ↓ MMP-2/-9 activity	[243]
Nordihydroguaiaretic acid (NDGA)	Human prostate cancer cell line (PC-3) (10–20 μM)	Nude mice s.c. or i.v. inoculated with 5 × 10^6^ PC-3 or luc-PC-3 cells and treated i.v. with 50–100 mg/kg of NDGA	↓ Cell migration by suppressing NRP1 expression ↓ FAK signaling pathway	[213]
*Stilbenes*				
Resveratrol	Human colon cancer cell lines (HCT116, RKO) (1–2–5 μM)		↓ EMT progression, NF-κB nuclear translocation and paxillin expression by targeting the β1-integrin receptor	[170]
	Human colon cancer cell lines (HCT116, SW480) (5 μM)		↓ FAK activity ↓ Cytoskeletal proteins	[214]
	Human colon cancer cell line (HT29) (50–100–150 μM)		↓ Talin expression ↓ FAK phosphorylation	[215]
	Human breast cancer cell line (MDA-MB-231) (1–10–25–50–100 μM)		↓ Cell migration ↓ Focal adhesion formation ↓ FAK phosphorylation ↑ Filopodia formation	[216]
	Human colon cancer cell lines (HCT116, RKO, SW480) (5 μM)		↓ NF-κB pathway ↓ FAK activity ↓ Vimentin production ↓ Slug nuclear factor activity ↑ E-cadherin expression	[171]
Resveratrol and Pterostilbene	Human cervical cancer cell lines (HeLa, CaSki, SiHa) (6.5–12–5–20–25–40–50–100–200 μM)		↓ MMP-2/-9 expression	[244]
Pterostilbene	Human breast cancer cell line (MCF-7) (5–10–20–30 μM)		↓ HRG-β1-mediated cell invasion, motility and cancer cell transformation through downregulation of MMP-9 activity	[245]
*Xanthones*				
Mangiferin	Human breast cancer cell lines (MDA-MB-231, BT-549, MCF-7, T47D) (75–150–300 μM)	SCID female mice s.c. inoculated with 2 × 10^6^ MDA-MB-231 cells and treated with 100 mg/kg of mangiferin by gavage	↓ MMP-7/-9 activity ↓ EMT progression ↓ Catenin pathway ↓ Tumor weight and progression ↑ Apoptosis induction ↑ E-cadherin expression	[168]
	Human prostate cancer cell line (LNCaP) (100–200–400 μM)		↓ TNF-α-induced MMP-9 expression by inhibition of NF-κB nuclear factor activity	[246]
	Human astroglioma cells (U87MG, U373MG, CRT-MG) (30–100–300 μM)		↓ MMP-7/-9 expression ↓ EMT progression	[247]
		C57BL/6 male mice inoculated in footpads with B16-BL6 cells and orally treated with 50–100–200 mg/kg of mangiferin	↓ MMP-1/-2/-9/-14 expression ↓ VLA-4/-5/-6 expression	[252]
	Human ovarian cancer cell lines (A2780, ES-2) (37.5–75–150–300 μM)	BALB/c female nude mice s.c. inoculated with 4 × 10^6^ cells and treated with 20–60 mg/kg of mangiferin	↓ MMP-2/-9 activity	[248]
α-Mangostin	Human pancreatic cancer cell lines (BxPC-3, MIAPaCa-2) (5–7.5–10–15 μM)		↓ MMP-2/-9 activity ↑ E-cadherin expression	[165]
Gartanin	Human glioma cell line (T98G) (3–10 μM)		↓ MMP-2/-9 activity	[249]
Cratoxylumxanthone C	Human lung cancer cell lines (A549), human liver cancer cell line (HepG2), human breast cancer cell line (MCF7) (7.15–15 μM)		↓ Cell migration ↓ FAK/MMP-2 pathway	[250]

Abbreviations: ↑, increase/upregulation; ↓, decrease/downregulation; *ADAM10*, ADAM metallopeptidase domain 10; AP-1, activator protein 1; ARM-1, regulating molecule-1; AXL, AXL receptor tyrosine kinase; c-Met, mesenchymal–epithelial transition factor; *CALM1*, calmodulin 1; CD24, cluster of differentiation 24; Cdc42, cell division cycle 42; c-Met, mesenchymal–epithelial transition factor; COX-2, cyclooxygenase-2; DNA, deoxyribonucleic acid; EGFR, epidermal growth factor receptor; EMT, epithelial–mesenchymal transition; *EPHB2*, ephrin receptor B2; ERK, extracellular signal-regulated kinase; FAK, focal adhesion kinase; FAS, apoptosis antigen 1; FRK, tyrosine-protein kinase FRK; GRB2, growth factor receptor-bound protein 2; GSK-3β, glycogen synthase kinase-3 beta; *HDAC4*, histone deacetylase 4; HER-2, human epidermal growth factor receptor 2; HRG-β1, heregulin β1; HSP27, heat shock protein 27; IFN-γ, interferon gamma; IGF-1R, insulin-like growth factor-1 receptor; IL, interleukin; INPPL1, inositol polyphosphate phosphatase-like 1; i.p., intraperitoneal; i.v., intravenous; JNK, c-Jun N-terminal kinase; Ki-67, nuclear protein Ki67; MAPK, mitogen-activated protein kinase; MEK, mitogen-activated protein kinase; MMP, matrix metalloproteinase; mRNA, messenger ribonucleic acid; MT1-MMP, membrane type 1 matrix metalloproteinases; mTOR, mammalian target of rapamycin; NF-κB, nuclear factor-kappa B; NNK, nicotine-derived nitrosamine ketone; NRP1, neuropilin-1; p21, activating factor-1/cyclin-dependent kinase inhibitory protein-1; p38, p38 mitogen-activated protein kinase; p130Cas, p130 Crk-associated substrate; PAI, plasminogen activator inhibitor-1; p-Akt, phosphorylated Akt; p-ERK1/2, phosphorylated extracellular signal-regulated kinase 1/2; p-FAK, phosphorylated FAK; PI3K, phosphoinositide 3-kinase; p-IκB, phosphorylated IκB kinase; PKC, protein kinase C; PMA, phorbol 12-myristate 13-acetate; PTEN, phosphatase and tensin homolog; PTPN6, tyrosine-protein phosphatase non-receptor type 6; PTPRK, protein tyrosine phosphatase receptor type K; Rac1, Ras-related C3 botulinum toxin substrate 1; Rho A, Ras homolog family member A; ROCK1, Rho-associated kinase 1; s.c., subcutaneous; *SEPP1*, selenoprotein P; SMAD, suppressor of mothers against decapentaplegic; SNAI1/Snail, snail family transcriptional repressor 1, zinc finger protein 1; SNAI2/Slug, snail family transcriptional repressor 2, zinc finger protein 2; Sp-1, transcription factor Sp1; Src, proto-oncogene tyrosine-protein kinase Src (or cSrc); STAT3, signal transducer and activator of transcription 3; TIMP-1/2, tissue inhibitor matrix metalloproteinases 1/2; TNF-α, tumor necrosis factor-alpha; TNK2, tyrosine kinase non-receptor 2; TWIST-1, twist-related protein 1; u-PA, urokinase-type plasminogen activator; VEGF, vascular endothelial growth factor; VLA-4/5/6, very late activation antigen-4/5/6; v-Src, tyrosine-protein kinase transforming protein Src; ZO-1, zonula occludens protein 1.

## 6. Discussion

The FAP is a complex biological structure that connects the outside with the inside of the cell, allowing the cell to detect and respond to signals from the ECM. The continuous turnover of the FAPs allows cell motility and cancer cell invasion. The process of invasion and, subsequently, of metastasis is allowed by the degradation of the ECM. The plaque needs different proteins to work properly, which are present in the ECM, cell membrane and cytoskeleton. The main proteins involved in cell invasion are MMPs, found in the ECM, and cadherins found at the cell membrane [253]. Specifically, MMPs degrade ECM proteins, while changes in cadherins expression levels contribute to the EMT, also important to start the cell’s invasion process [254]. The FAP is also fundamental for cell survival thanks to the presence of multiple protein kinases, such as Akt, mTOR, PI3K and MAPKs, that start pro-survival signaling cascades [255]. Due to their key role in cancer cell migration and invasion, MMPs and components of FAPs are often upregulated in cancer and are thus potential targets for cancer therapy. In this regard, several studies have assessed the effects of polyphenols on FAPs and MMPs. Polyphenols are a large group of organic compounds found in plant-based food and beverages, classified into two main classes, flavonoids and non-flavonoids based on their chemical structure [256]. They are known for many beneficial properties like anticancer, anti-inflammatory, antioxidant and anti-aging properties.

This review provided a wide picture of the multifaced effects of polyphenols on FAPs in a tumorigenic context. In fact, polyphenols have been reported to inhibit enzymatic activity and/or expression of MMPs, specifically -2 and -9, and to promote the switch from mesenchymal to epithelial cadherins, thus inhibiting EMT [257,258]. Moreover, different polyphenols have shown the capacity to block FAK phosphorylation and the downstream pathway [259]. In some cases, polyphenols also showed the ability to block the formation of invadosomes, thus minimizing cells movement. 

One of the biggest problems with the use of polyphenols in the medical field is their poor bioavailability and low concentration in the bloodstream once administered orally [260]. This obviously has a strong impact on the effective dose that is delivered to cancer cells. One way to counteract this problem could be to combine several polyphenols with each other, thereby increasing their overall concentration at the cellular level and adding together the effects of the individual compounds [261,262]. Dietary variables can also impact the bioavailability of polyphenols, in addition to endogenous factors. The release of polyphenols can be influenced by a specific food matrix, and the composition and structure of polyphenols can be changed during food preparation [263]. Due to these factors, plasma only contains nano- or micromolar amounts of polyphenols and their metabolites (0–4 µM following an ingestion of 50 mg of aglycone equivalents) [264]. This is due to the fact that polyphenols are stably bound inside the food in which they are contained, and therefore, it is already difficult to break them apart and make them soluble. In addition, there are multiple enzymes produced by the intestinal microbiota that can metabolize these compounds, and only a very small amount of them reaches the bloodstream. The bioavailability of polyphenols also varies depending on the class, with a well-defined hierarchy: phenolic acids > isoflavones > flavonols > catechins > flavanones/proanthocyanidins > anthocyanins [265]. For all these reasons, the doses of polyphenols provided in oral treatments in in vivo experiments must be higher than those used in vitro. It should be also considered that in addition to the difficulties present at the level of intestinal absorption and metabolism, in in vivo models, there are much more complex cellular mechanisms and interactions than those present in stabilized cell cultures. 

Despite the huge advances made in recent years in the research and analysis of the anticancer effects of polyphenols, the main problem of their low bioavailability remains unresolved. The research so far mainly focused on in vivo and in vitro models, and few clinical trials have started to investigate the effects of polyphenols as anticancer drugs in humans. Future research may focus on strategies aimed at increasing polyphenols’ bioavailability. Recently, several strategies are being considered. One of these may be the use of different classes of polyphenols joined to certain anticancer drugs [266]. This strategy, however, is not free from risks since the interaction between polyphenols and drugs can induce the occurrence of side effects, and the mechanisms of action are not yet fully clear. Another method can be the use of nanotechnology to produce lipid nanoparticles to be employed as “containers” for polyphenols, so as to allow them to cross the intestinal barriers and reach the blood circulation in larger quantities [267,268,269,270].

Considering the studies reported so far, polyphenols could have a potential as adjuvants for chemo- and radiotherapy because of their capacity to inhibit key proteins used by cancer cells to invade the ECM and promote cell survival.

## 7. Conclusions

In conclusion, this review provides a comprehensive understanding of the diverse effects of polyphenols on FAPs in a tumorigenic context. Polyphenols exhibit promising abilities to inhibit crucial proteins involved in cancer progression, such as MMPs, and facilitate the reversal of EMT, thus affecting cancer cell invasion. However, their clinical application is hindered by challenges related to their poor bioavailability and low bloodstream concentration after oral administration. Strategies to enhance polyphenol bioavailability, such as combination therapies and nanotechnology-based delivery systems, are being explored. Despite these challenges, polyphenols hold potential as adjunctive therapies to conventional cancer treatments due to their ability to target key mechanisms involved in cancer progression. Future research efforts should prioritize the development of effective strategies to improve polyphenol bioavailability and elucidate their mechanisms of action in clinical settings.

## Data Availability

Not applicable.
